# Academic Resilience and Behavioral Patterns in a Digital Problem-Solving Task: Evidence from Process Data

**DOI:** 10.3390/jintelligence14100252

**Published:** 2026-10-10

**Authors:** Yunhan Sun, Jinxi Ji, Jinxin Guo, Xin Xu

**Affiliations:** School of Science, Minzu University of China, Beijing 100081, China

**Keywords:** academic resilience, digital problem solving, process data, action sequence analysis, behavioral profiles, PISA

## Abstract

Digital problem-solving assessments provide process data that can reveal how students behave beyond final achievement. Previous research has shown substantial individual differences in problem-solving behavior. Academic resilience commonly refers to strong academic performance despite socioeconomic disadvantage, but little is known about how academically resilient students behave during problem solving. This study examined whether socioeconomically disadvantaged students classified as academically resilient showed distinctive behavioral patterns in the Programme for International Student Assessment (PISA) 2012 TICKET task. Academic resilience was defined using socioeconomic disadvantage and mathematics achievement, and propensity-score matching (PSM) was used to improve comparability on selected background characteristics. Process data were analyzed as action sequences to identify recurring behavioral profiles. Results showed that resilient students tended to follow longer action sequences and had higher backtracking proportions, whereas differences in timing indicators were not statistically significant. These findings suggest that mathematics-based academic resilience is associated with differences in the organization of task behavior.

## 1. Introduction

Socioeconomic disadvantage remains strongly associated with students’ academic performance. In PISA 2022, socioeconomically advantaged students outperformed their disadvantaged peers in mathematics by 93 points on average across OECD countries (OECD, 2023). Similar socioeconomic differences have also been observed in computer-based problem solving. Family background is therefore also related to students’ performance in complex and interactive tasks (OECD, 2014a). Nevertheless, socioeconomic disadvantage does not fully determine students’ academic outcomes. Some students from disadvantaged backgrounds still attain relatively high levels of achievement despite adverse conditions (Aydın & Erdem, 2023; Ng, 2020). These students are commonly described as academically resilient (OECD, 2011). Academic resilience has received growing attention in international large-scale assessment research. Since the OECD report Against the Odds (OECD, 2011), subsequent studies have increasingly examined the factors associated with resilient academic performance among socioeconomically disadvantaged students (Sandoval-Hernández & Białowolski, 2016).

An increasing number of studies have focused on identifying the characteristics associated with academic resilience. García Crespo et al. (2019) examined individual and family factors that distinguish academically resilient students from other disadvantaged students. Agasisti et al. (2021) further showed that school conditions, including disciplinary climate and instructional support, were associated with resilient academic performance. More recently, K. C. Cheung et al. (2024) used machine-learning methods to identify a broader set of student, family, and school characteristics related to academic resilience in PISA 2022. These studies have clarified who academically resilient students are and which factors are associated with their academic success (Zheng et al., 2024).

Academic resilience is typically identified from socioeconomic background and achievement outcomes (Agasisti et al., 2018). However, little is known about how academically resilient students behave when they encounter a challenging task. Computer-based assessments provide an opportunity to examine this behavior because students’ actions and timing are recorded during task completion (Greiff et al., 2015). Such process data can be used to characterize the ordering and timing of students’ interactions with a task (Greiff et al., 2015; Ulitzsch et al., 2021). Linking these records to academic-resilience classifications makes it possible to examine whether disadvantaged students with different achievement outcomes exhibit different patterns of problem-solving behavior. The present study uses action sequences and timing data from the Programme for International Student Assessment (PISA) 2012 TICKET task to examine behavioral profiles and their associations with mathematics-based academic resilience among socioeconomically disadvantaged students.

## 2. Literature Review

### 2.1. Academic Resilience in International Large-Scale Assessments

Resilience has been examined across psychological, ecological, and social research (Adger, 2000; Luthar et al., 2000). In education, academic resilience refers to successful academic functioning despite conditions associated with an increased risk of underachievement (K. C. Cheung et al., 2024; Rudd et al., 2021). Its defining elements are adversity and positive adaptation (Masten, 2001; Windle, 2011). Adversity increases the likelihood of unfavorable outcomes without making them inevitable, while positive adaptation denotes successful functioning within that risk context (Luthar et al., 2000). Academic resilience therefore requires academic success to be evaluated in relation to the adverse circumstances students experience (Ye et al., 2021). This perspective motivates research on the personal and environmental resources associated with successful performance under disadvantage (Borman & Overman, 2004).

Achievement-based definitions are commonly used in international large-scale assessment research (Sandoval-Hernández & Białowolski, 2016; Ye et al., 2021). In PISA, socioeconomic disadvantage is frequently defined as placement in the bottom 25% of the index of economic, social and cultural status (ESCS) within each country or economy. The achievement criterion can then be specified from a national or international perspective. Under the within-country definition, resilient students meet this background criterion and attain mathematics scores at or above the national 75th percentile (OECD, 2023). K. C. Cheung et al. (2024) adopted an international definition that retained the country-specific ESCS criterion and identified students whose mathematics scores reached the 75th percentile across 79 countries and economies. The former identifies high achievement relative to national peers, while the latter applies a common achievement threshold across participating education systems (K. C. Cheung et al., 2024; Ye et al., 2021). Proficiency-based definitions assess success against specified skill levels. For example, Agasisti et al. (2018) classified students in the bottom national ESCS quartile as resilient when they attained at least Level 3 in all three PISA domains: reading, mathematics, and science. These approaches operationalize academic success against different reference standards within a shared outcome-based framework, providing a basis for examining the characteristics and educational conditions associated with resilient performance (Agasisti et al., 2018; K. C. Cheung et al., 2024).

Research within this framework links resilient academic performance to both individual characteristics and learning environments. Greater engagement in academic activities and positive attitudes toward school have been associated with academic success among disadvantaged students (Borman & Overman, 2004; Finn & Rock, 1997). Mathematics self-efficacy and confidence are also associated with resilient mathematics outcomes (K.-c. Cheung, 2017; García-Crespo et al., 2022; Pitsia & Perkins, 2025). Evidence on school conditions complements these individual-level findings. Using PISA data, Agasisti et al. (2021) found that successful disadvantaged students attended schools with more favorable disciplinary climates and additional instructional time in key subjects. K. C. Cheung et al. (2024) examined these domains within a framework encompassing psychological factors and social and environmental conditions. The analysis of PISA 2022 identified 35 features associated with academic-resilience classifications. Resilient students tended to report higher self-efficacy, lower truancy, and more positive future aspirations. Together, these findings support examining resilient academic outcomes in relation to students’ beliefs and participation in learning, alongside the educational conditions in which learning occurs (Agasisti et al., 2021; Borman & Overman, 2004; K. C. Cheung et al., 2024).

The associations with engagement and school participation motivate closer examination of students’ behavior within specific academic tasks. However, the contextual features examined by K. C. Cheung et al. (2024) were drawn from student and school questionnaires. These measures describe beliefs, reported experiences, and learning conditions, but provide limited information about the ordering and timing of actions during task completion. Process records provide complementary evidence by documenting students’ interactions with a task as they occur (Q. He et al., 2021). Linking these records with achievement-based classifications makes it possible to examine whether resilient and non-resilient students differ in how they organize their problem-solving behavior. This extends the study of resilience-related characteristics to observable patterns of task activity within a socioeconomically disadvantaged population.

Behavioral research has linked academic resilience with classroom participation and school engagement. Classroom observations showed that teacher-identified resilient pupils spent more time in instructional interactions with teachers (Padron et al., 1999). Questionnaire research also found a positive association between academic resilience and school engagement (Romano et al., 2021). These studies describe students’ participation in learning activities, but provide limited information about how their actions are organized within individual tasks. Examining the sequence and timing of task actions can complement this evidence by describing how students approach problem solving.

### 2.2. Problem-Solving Behavior in Digital Tasks

Problem solving requires pursuing a goal when a solution procedure is not immediately available (Greiff et al., 2013; OECD, 2014a). Process accounts connect understanding a problem with selecting actions and evaluating their consequences (Dörner & Funke, 2017; Fischer et al., 2012). The PISA framework similarly encompasses problem representation, planning, execution, monitoring, and reflection in digital tasks (OECD, 2014a). These processes vary in their relevance across tasks because problem structures and available information differ (Greiff et al., 2013; Jonassen, 2000). The course of activity may also change as a solution develops. Information obtained during execution can lead students to reconsider an earlier decision (OECD, 2014a). Problem-solving behavior therefore involves coordinating successive actions in relation to task requirements.

Sequence length provides one description of this activity. Some studies have found that successful respondents engage more extensively with task-relevant functions. For example, Xiao et al. (2021) reported more frequent use of relevant tools and greater task engagement among correct-response groups. Yet a larger number of actions does not consistently indicate better performance. Among students who applied the same exploratory procedure, Stadler et al. (2019) found no significant differences in total action counts or working time between successful and unsuccessful groups. The groups nevertheless differed in how their actions were organized. Similarly, Q. He et al. (2021) showed that few high-performing respondents consistently solved digital problems using the minimum number of actions. Sequence length therefore describes the amount of activity, but provides limited information about how that activity contributes to task completion.

Repeated actions and returns provide a second behavioral dimension. Their function varies with the task state, preceding actions, and subsequent choices (Chen, 2020; Kroehne & Goldhammer, 2018; K. D. Wang et al., 2023). Eichmann et al. (2020) identified successful patterns involving both economical execution and repeated goal-relevant actions. The authors interpreted the latter pattern as double-checking relevant information. Other studies have also documented heterogeneous behavioral patterns within performance groups (Ulitzsch et al., 2021; Xiao et al., 2021). These findings indicate that the frequency of returns or repetitions provides only partial evidence about problem-solving quality. Their interpretation depends on the actions surrounding them and the task outcome.

Temporal indicators provide an additional description of the response process. Time on task can reflect greater cognitive investment, while faster performance can also reflect greater fluency (Goldhammer et al., 2014; Vörös & Rouet, 2016). Goldhammer et al. (2014) showed that the association between time and success varied with task difficulty and individual skill. Vörös and Rouet (2016) likewise found that relationships among response time, action count, and task outcome differed across digital problem-solving tasks. More fine-grained timing information can reveal further variation. Studies combining action order with action-level timing have identified different response processes among respondents working on the same task (Ulitzsch et al., 2021). Related research on digital navigation also shows that performance is associated with the joint organization of navigation paths and time allocation (Q. He et al., 2023). Accordingly, total duration, action intervals, and the timing of particular events capture different aspects of task behavior. Their interpretation depends on the actions performed and their location within the response process.

In this study, academic resilience is treated as an outcome-based classification of successful achievement under socioeconomic disadvantage. Adaptive problem solving and self-regulation provide theoretical perspectives on how students respond to changing task demands and monitor ongoing activity (Greiff et al., 2017; Veenman et al., 2006; Zimmerman, 2000). These processes are not directly measured by the process logs. Adaptive persistence is therefore used only as a possible interpretation of observed patterns involving continued engagement and revision, not as a separate construct or a mechanism underlying academic resilience.

### 2.3. Action Sequence Analysis in Computer-Based Assessment

Computer-based assessments record response processes as ordered, time-stamped events. Action sequences preserve the order in which respondents interact with a task and therefore provide information that is not contained in final scores or aggregate indicators alone (Anghel et al., 2024; Bergner & von Davier, 2019; S. He & Cui, 2025). However, raw log files are highly variable in length, event type, and task structure. Their analysis requires decisions about how recorded events are represented as meaningful behavioral information (Kroehne & Goldhammer, 2018; K. D. Wang et al., 2023). Recent reviews therefore distinguish the collection of process data from feature construction and subsequent interpretation. This distinction is particularly important for action sequences because different representations preserve different aspects of the response process (Anghel et al., 2024; K. D. Wang et al., 2023).

One approach represents response processes through selected actions or short subsequences. *N*-gram methods retain the local order of adjacent events and have been used to identify action patterns associated with successful and unsuccessful responses (Q. He & von Davier, 2016). Feature-based analyses can also combine selected action patterns with timing variables. Using the PISA 2012 TICKET unit, Qiao and Jiao (2018) showed that such features can be used both to classify performance and to identify groups with different response processes. Sequential features have also been linked to respondents’ background characteristics. For example, Liao et al. (2019) identified process patterns associated with employment-related characteristics in Programme for the International Assessment of Adult Competencies (PIAAC). These approaches provide interpretable summaries of selected parts of a response process. Their focus on local events, however, retains only part of the dependence structure contained in a complete action path (Tang et al., 2021; Xiao et al., 2021).

A complementary approach treats the complete sequence as the unit of analysis. Sequence-distance methods quantify similarities or differences between ordered action paths. Hao et al. (2015) used edit distance to compare observed sequences with a reference sequence, while Q. He et al. (2021) applied the longest common subsequence (LCS) to compare respondents’ action paths with expert-defined solutions across multiple PIAAC tasks. Greater similarity to the reference paths was associated with higher problem-solving proficiency, although high-performing respondents did not consistently use the minimum number of actions. Sequence similarity can also be defined directly between respondents. Ulitzsch et al. (2021) combined action-sequence similarity with action-level timing and used graph-based clustering to identify groups with common response processes. In PISA 2018 digital reading, Q. He et al. (2023) similarly combined navigation paths and time allocation to identify distinct navigation profiles. These studies show that whole-sequence analysis can represent similarities in behavioral organization even when respondents differ in the number or timing of individual actions.

Other approaches describe sequence structure through latent representations, states, or subprocesses. Tang et al. (2021) used sequence-to-sequence autoencoders to transform variable-length action sequences into numerical features that retained information about task outcomes and respondent characteristics. Xiao et al. (2021) modeled response processes as transitions among latent states and identified systematic differences in state use across performance groups. At a different level of granularity, Z. Wang et al. (2023) segmented complete response processes into data-driven subtasks based on changes in action predictability. Model-based approaches have also integrated the temporal dimension more directly. Event-history and continuous-time models incorporate both event occurrence and timing (Chen, 2020; Chen et al., 2019), while Fu et al. (2024) jointly modeled action sequences and action-level response time. These developments illustrate that action-sequence information can be represented at multiple levels, from observed paths to latent states and continuous processes. The choice of representation determines which aspects of behavior remain available for interpretation.

Sequence analysis has increasingly been used to examine differences between groups of respondents. Action-sequence features can contain information about demographic and cognitive characteristics beyond that available from final item scores (Zhang et al., 2024). Sequence-based studies have also identified differences associated with proficiency, age, gender, socioeconomic background, and other respondent characteristics (Q. He et al., 2021, 2023; Liao et al., 2019). Within the PISA 2012 TICKET unit, feature-based clustering and biclustering have revealed substantial heterogeneity in students’ response processes (Qiao & Jiao, 2018; Xu et al., 2024). Existing research therefore provides a substantial basis for using action sequences to study behavioral heterogeneity in computer-based assessment. The specific connection between such behavioral profiles and achievement-defined academic resilience within a socioeconomically disadvantaged population, however, has received much less attention. This gap motivates the comparison developed in the present study.

### 2.4. Current Study

The present study examines the association between academic resilience and the organization of problem-solving behavior among socioeconomically disadvantaged students. Process data from item CP038Q02 in the PISA 2012 *TICKET* unit are used. The item requires students to make successive choices within a simulated ticket-purchasing environment. Students can cancel their selections, return to the initial state, and make new selections before confirming a purchase (OECD, 2014a). Previous research on TICKET process data has identified substantial variation in students’ response processes (Qiao & Jiao, 2018; Xu et al., 2024). The interface therefore permits more than one observable response path and allows earlier selections to be abandoned before a final purchase is confirmed. This structure makes CP038Q02 suitable for examining variation in sequence length, cancellation, return, subsequent choice revision, and the timing of these actions.

Accordingly, this study examines whether behavioral-profile membership is associated with academic resilience among socioeconomically disadvantaged students. In this study, academic resilience is operationalized using socioeconomic background and mathematics achievement. The study also compares action-sequence and timing characteristics across the identified profiles and between the two groups. By linking this outcome-based classification with task-level process data, the study extends existing research to the organization of students’ behavior during digital problem solving.

Specifically, the following research questions were addressed:

RQ1 Can action sequences in the TICKET task be grouped into interpretable behavioral profiles among socioeconomically disadvantaged students?
RQ2 Are these behavioral profiles associated with academic resilience status?
RQ3 How do action-sequence and timing characteristics vary across the identified behavioral profiles and between the resilient and non-resilient groups?

## 3. Materials and Methods

### 3.1. Data Source and Task

The data were drawn from PISA 2012, which assessed nationally representative samples of 15-year-old students in participating education systems. PISA 2012 used a stratified two-stage probability sampling design. Schools enrolling PISA-eligible students were first sampled, generally with probabilities related to school size, and eligible students were subsequently sampled within participating schools (OECD, 2014b). The present study used data from Australia, Singapore, Turkey, and the United States. The analysis was restricted to socioeconomically disadvantaged students with linkable process records. A matched analytical sample was then constructed. The findings are therefore interpreted as sample-specific associations, not nationally representative estimates.

Two components of the PISA 2012 database were linked for the analysis: student-background data and computer-based process logs. The background data provided country and student identifiers, the index of ESCS, mathematics plausible values, and variables describing demographic, family, and learning conditions. The process logs recorded the ordered events generated while students interacted with the computer-based task, together with task states and time stamps. The two sources were linked using country and student identifiers.

**Figure A1:**
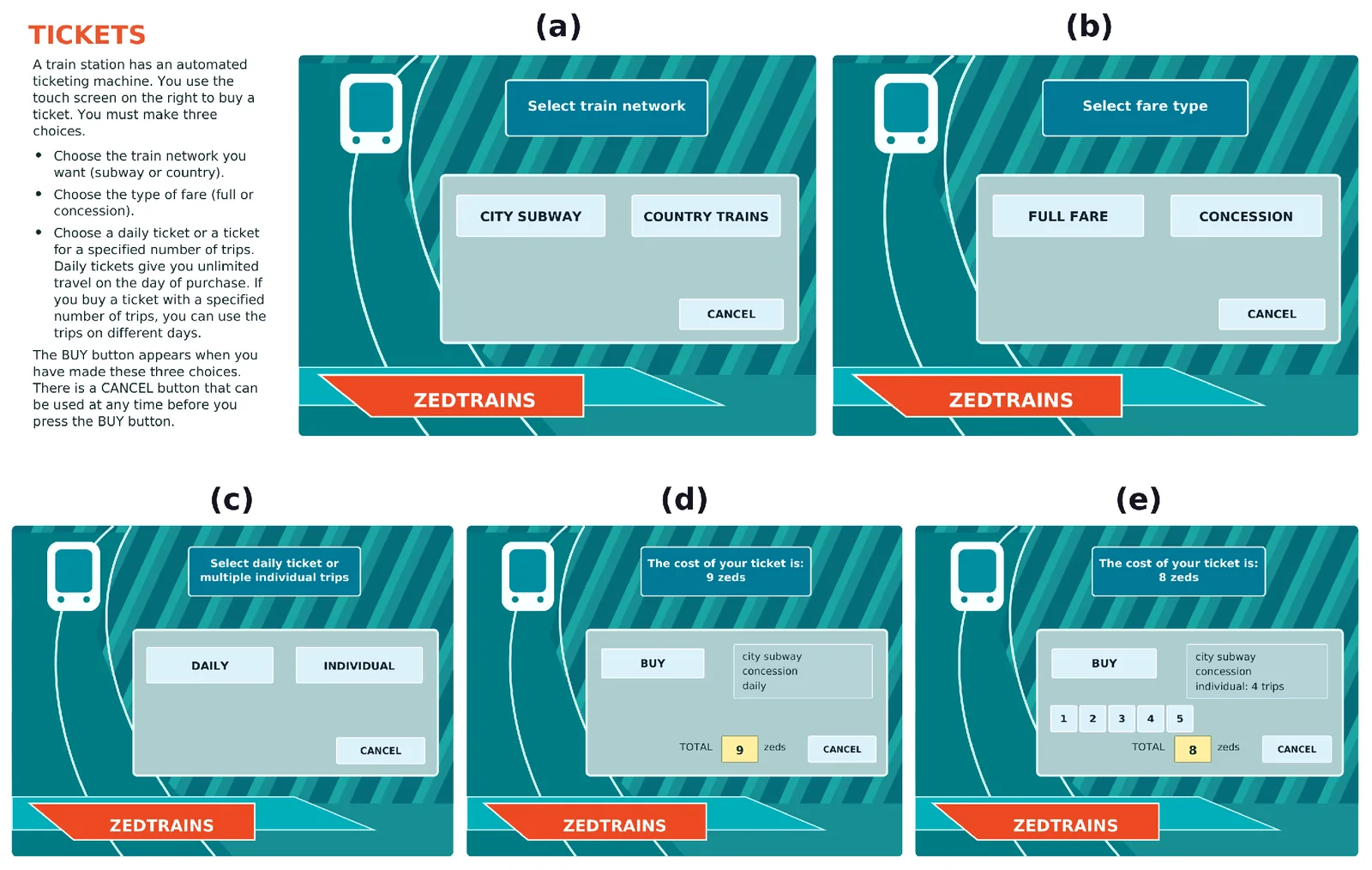
Example screens from the PISA 2012 TICKET task interface: (**a**) transport network selection; (**b**) fare type selection; (**c**) ticket type selection; (**d**) number of trips selection; (**e**) purchase confirmation.

The behavioral analysis focused on item CP038Q02 in the PISA 2012 *TICKET* unit. The item presented a simulated ticket-purchasing environment in which students made a sequence of selections concerning the transport network, fare type, ticket type, and number of trips (OECD, 2014a). A correct solution required selecting the country-train network, full fare, an individual ticket, and two trips, followed by purchase confirmation. CP038Q02 permits multiple observable response paths before purchase confirmation. Students can revise earlier choices through cancellation and return, producing variation in action order, sequence length, and timing. Task success was defined using the official dichotomous item score, that is, a score of 1 indicated a correct response and 0 indicated no credit. Special missing-value codes were excluded from analyses requiring an observed item outcome. Purchase confirmation or item termination alone was not treated as evidence of correctness. Example screens from the TICKET interface are provided in Appendix A Figure A1.

### 3.2. Resilience Definition and Sample Eligibility

Resilience status was defined before restricting the background sample to students represented in the TICKET process data. The initial four-country sample contained 29,853 students. Classification required observed ESCS and PV1MATH, leaving 29,350 students. Within each country, students whose ESCS was at or below the country-specific 25th percentile were classified as socioeconomically disadvantaged. The percentiles were calculated from the eligible national samples without survey weights. The resulting ESCS thresholds were −0.390 for Australia, −0.915 for Singapore, −2.280 for Turkey, and −0.490 for the United States. This criterion identified 7429 disadvantaged students.

Among disadvantaged students, those with PV1MATH scores of 482 or higher were classified as academically resilient, whereas those below 482 were classified as non-resilient. The fixed mathematics threshold follows the PISA 2012 criterion reported by Yang Hansen et al. (2025). Thus, socioeconomic disadvantage was defined relative to each national context, whereas the mathematics criterion was held constant across countries. Both criteria were established before linkage to the TICKET data and were not recalculated within the process-data sample or after matching.

**Figure 1:**
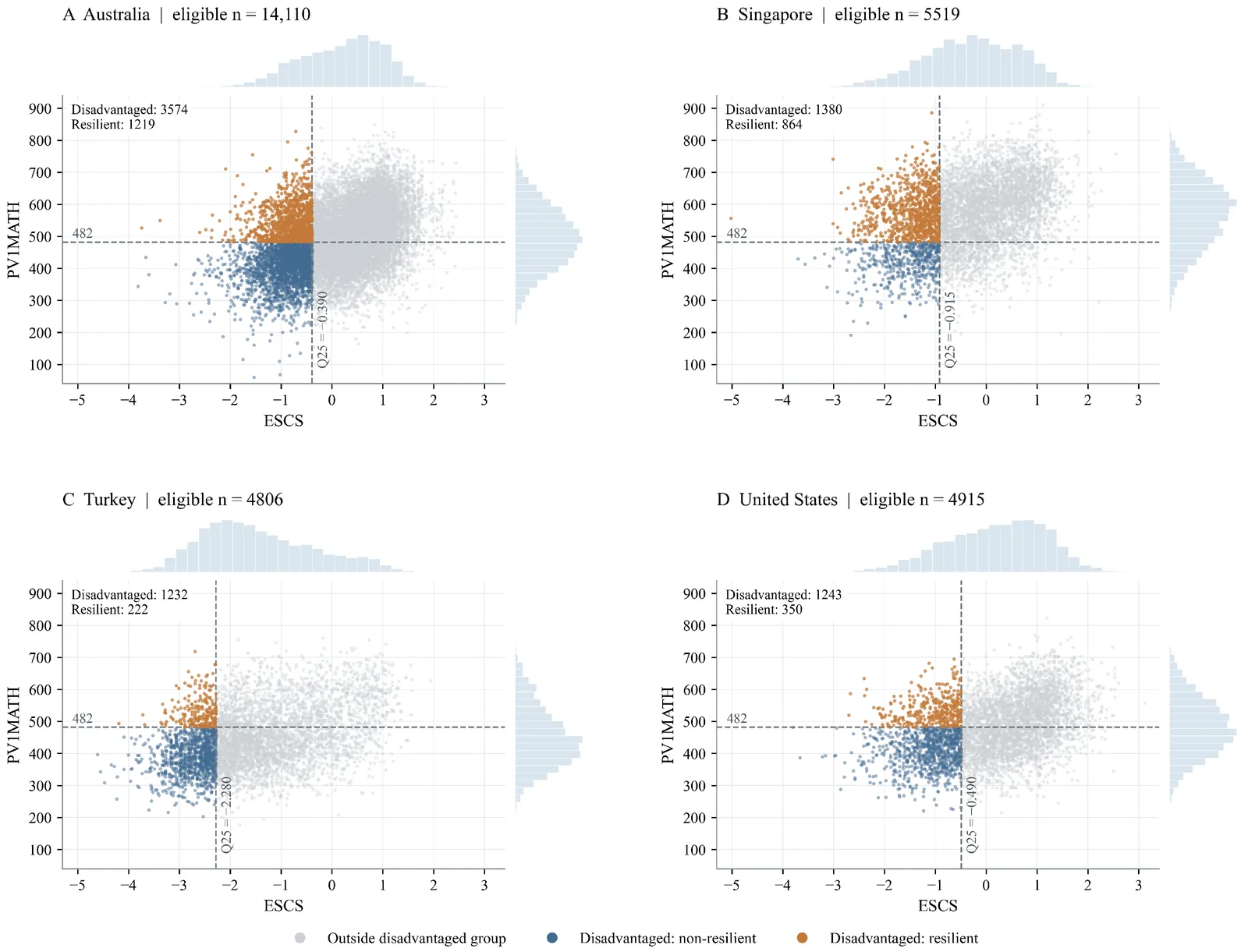
Operational definition of academic resilience in the four national source samples. The vertical dashed line marks the country-specific ESCS 25th percentile, and the horizontal dashed line marks the fixed mathematics threshold of PV1MATH = 482. Orange denotes disadvantaged students classified as resilient, blue denotes disadvantaged non-resilient students, and gray denotes students outside the disadvantaged group. Classification was completed before linkage to the TICKET process data and before matching.

Figure 1 illustrates the classification in the four national source samples. Linkage to the TICKET process data identified 929 disadvantaged students, including 308 resilient and 621 non-resilient students. After the missing-data procedure described in the next subsection, 20 completed datasets were generated, each containing the same 708 students eligible for matching, including 274 resilient and 434 non-resilient students. Matching and balance assessment were conducted separately in all 20 datasets. One of the 20 completed datasets was randomly selected for the principal process-data analyses; this procedure selected the seventh dataset, which yielded 220 matched pairs (N=440). Sample construction is summarized in Table 1, and the matching procedure is described in Section 3.4.

### 3.3. Covariates and Missing-Data Handling

Seven covariates were used to characterize student background and learning conditions: gender (ST04Q01), immigrant background (IMMIG), family structure (FAMSTRUC), perceived teacher support in mathematics classes (TEACHSUP), student–teacher relations (STUDREL), mathematics teacher support (MTSUP), and weekly mathematics instructional time (MMINS). The first three variables were treated as categorical and the remaining four as numeric. TEACHSUP and MTSUP were retained as separate indices in accordance with the PISA 2012 documentation (OECD, 2014b).

These variables were selected from the broader set of student-level covariates considered by Yang Hansen et al. (2025). They represent demographic background, family context, classroom support, and instructional exposure. In this paper, mathematics-related affective, motivational, and self-belief variables were not included because they are conceptually proximal to the resilience classification and to the behavioral processes examined in this study. Mathematics performance was not included because it formed part of the resilience definition. ESCS defined socioeconomic disadvantage and was used in the imputation models, but it was not included as an additional matching covariate.

Missing covariate data were distinguished between planned missingness associated with the PISA questionnaire design and residual ordinary missingness. Examination of questionnaire-form-specific missingness identified TEACHSUP, STUDREL, and MTSUP as planned-missing variables in form 1 and MMINS in form 2. Missing values in these prespecified variable–form combinations were designated for imputation. Other missing covariate values were treated as ordinary missingness and were not imputed.

Among the 929 disadvantaged students linked to the TICKET process data, 317 values each for TEACHSUP, STUDREL, and MTSUP and 323 values for MMINS were designated for imputation. After imputation of planned-missing cells, students with remaining missing values on any of the seven covariates were excluded. This removed 221 students and left the same 708 students in each completed dataset, including 274 resilient and 434 non-resilient students.

Twenty completed datasets were generated using an iterative Bayesian-ridge-assisted donor imputation procedure (van Buuren & Groothuis-Oudshoorn, 2011). For each target variable, the imputation model included the other six covariates together with country, ESCS, PV1MATH, questionnaire form, and resilience status. Detailed identification rules, implementation settings, and diagnostics are provided in Appendix C, while covariate balance and collinearity diagnostics are reported in Appendix B.

Collinearity among the seven covariates was assessed before matching using generalized variance inflation factors (GVIFs) (Fox & Monette, 1992). For a term occupying columns *J* of the design correlation matrix *R*,

(1) $${\mathrm{GVIF}}_{J}=\frac{\det\left(R_{JJ}\right)\det\left(R_{-J,-J}\right)}{\det(R)},\;{\mathrm{aGVIF}}_{J}={\mathrm{GVIF}}_{J}^{1/(2{\mathrm{Df}}_{J})}.$$

GVIFs were calculated separately in each of the 20 completed datasets. Country was not included in this seven-covariate diagnostic. Raw and dimension-adjusted GVIFs are summarized across completed datasets in Appendix B. No covariate was removed on the basis of these diagnostics.

### 3.4. Propensity-Score Matching and Final Analytical Sample

Because the data are observational, propensity-score matching (PSM) was used to improve comparability between resilient and non-resilient students on observed background characteristics (Rosenbaum & Rubin, 1983; Stuart, 2010). Resilience status was an outcome-based classification rather than an experimentally assigned treatment. Accordingly, PSM was used as a design-stage balancing procedure to construct a non-resilient comparison group with a more similar distribution of selected observed demographic, family, and learning-context characteristics. Its inferential purpose was to reduce observed compositional differences between the two groups before comparing their process-data outcomes, not to estimate a causal effect of resilience status.

Propensity scores were estimated separately in each of the 20 completed datasets using unweighted logistic regression. Resilience status was the dependent variable. The model included the seven covariates described above together with country indicators. The propensity score for student *i* was defined as

(2) $$e\left(X_{i}\right)=P\left(T_{i}=1|X_{i}\right),$$

where $T_{i}=1$ denotes resilient status and $X_{i}$ contains the matching covariates. This approach relies on sufficient overlap in the estimated propensity scores and on the adequacy of the observed covariates and propensity-model specification. Matching can reduce imbalance in measured characteristics, but it cannot eliminate residual imbalance or differences in unmeasured characteristics. The subsequent behavioral contrasts are therefore interpreted as associations within the matched sample.

Within each completed dataset, pooled one-to-one Mahalanobis nearest-neighbor matching was performed without replacement and subject to a propensity-score caliper. The caliper was set to 0.25 times the standard deviation of the estimated propensity probabilities in the matching pool, following the specification used by Yang Hansen et al. (2025). The caliper restricted matching to students with sufficiently similar estimated propensity scores. Resilient students were processed in decreasing propensity-score order. Among eligible non-resilient students within the caliper, the student with the smallest Mahalanobis distance was selected. Students without an available match within the caliper were left unmatched. Country was included in the matching variables rather than imposed as an exact matching restriction; cross-country pairs were therefore permitted.

Matching was conducted separately in all 20 completed datasets. The number of retained pairs ranged from 210 to 222, with a mean of 216.35 pairs. One completed dataset was randomly selected for the principal process-data analyses; this procedure selected the seventh dataset. The selected dataset yielded 220 matched pairs (N=440), including 195 within-country pairs and 25 cross-country pairs. The subsequent process-data analyses are therefore conditional on this selected completed dataset.

Covariate balance was evaluated before and after matching using standardized mean differences (SMDs). For a numeric covariate *X*,

(3) $$\mathrm{SMD}=\frac{{\bar{X}}_{1}-{\bar{X}}_{0}}{\sqrt{(s_{1}^{2}+s_{0}^{2})/2}}.$$

For categorical variables, SMDs were calculated for category indicators and summarized by the maximum absolute value within each term. Values below |SMD|=0.1 were used as a practical reference for good balance (Austin, 2009). Balance assessment was conducted separately in all 20 completed datasets, and the mean and observed range of each term’s absolute SMD are reported in the Appendix B.

### 3.5. Action Coding and Verification

**Figure 2:**
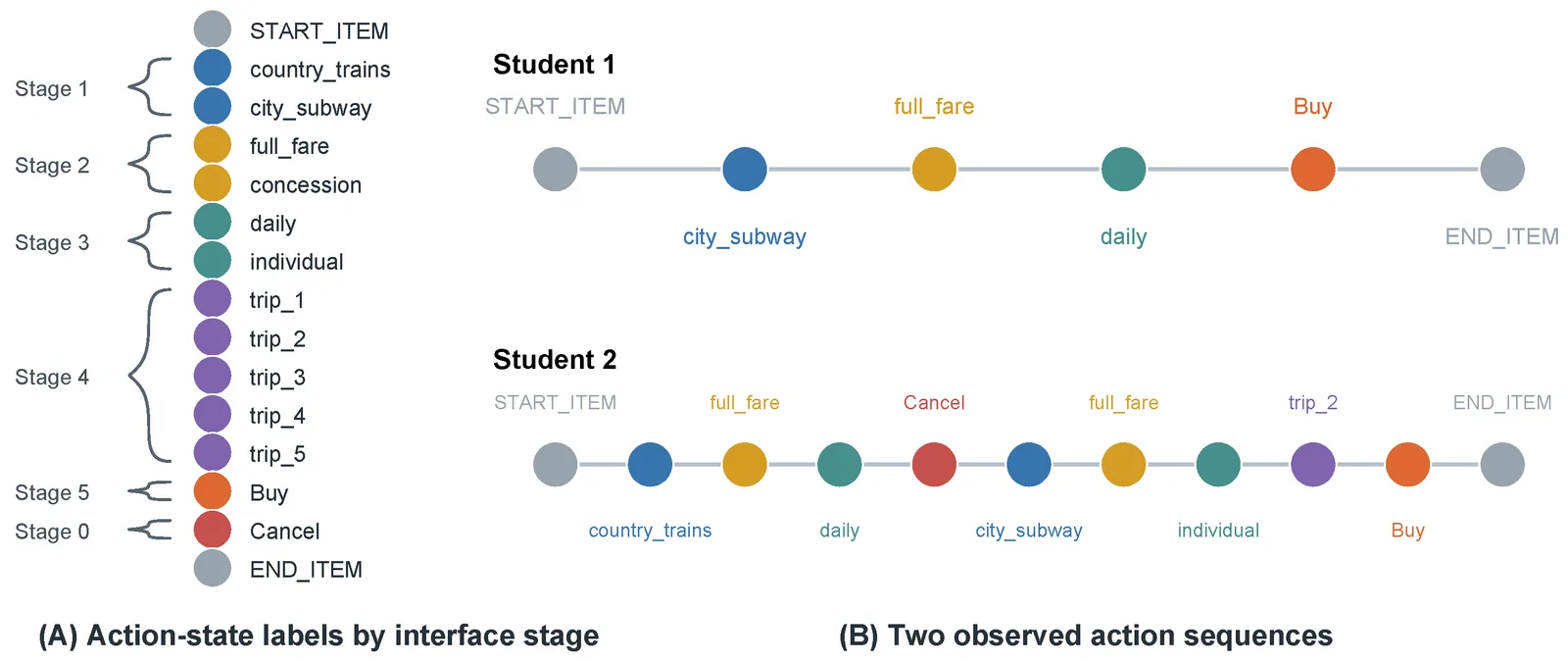
Transformation of TICKET process logs into student-level action sequences. Action-state labels are organized by their positions in the task interface, and the two examples illustrate observed response paths.

Raw state strings in the TICKET logs were mapped to descriptive action-state labels shown in Table A1. CP038Q02 uses a fixed interface with a finite set of observable selections and operations. The 15 action-state labels cover all action and state types recorded in the analytical logs for this item, including the administrative markers START_ITEM and END_ITEM. These labels were not derived from observed behavioral patterns or defined through a data-driven classification procedure. The mapping was based on observable states and operations in the TICKET interface and was checked against the official task documentation (OECD, 2014a). The mapping therefore converted the recorded interface-state strings into human-readable labels corresponding to the fixed task interface. These labels identify task actions and administrative markers and have no ordinal meaning. For example, the sequence city_subway–concession–daily–Buy represents the selection of a city-subway concession daily ticket followed by purchase confirmation. The Cancel action clears the current selections and returns the interface to the initial network-selection screen. Figure 2 illustrates the conversion from raw logs to action sequences. Sequence similarity and clustering were based on these action-state labels. All unique state strings observed in the analytical logs were accounted for by this mapping, and no observed state remained unmapped or required discretionary recoding.

Actions such as cancellation, return, and repeated selection are treated as task-level behavioral indicators whose meaning is interpreted in relation to their position and surrounding actions within the complete response sequence.

### 3.6. Behavioral Measures and Sequence Similarity

Five primary behavioral and timing measures were used in the paired comparisons. Sequence length was defined as the number of actions in the retained analytical sequence. First-response time characterized the time taken before the first task action. Mean action interval summarized the average temporal spacing between actions during task completion, and total task duration represented the overall time spent on the task. To account for multiplicity across the five primary behavioral and timing comparisons, the corresponding Wilcoxon *p* values were adjusted using the Holm procedure.

Additionally, we employed the *backtracking proportion* (BP) to summarize returns to the beginning of the task relative to the number of observed action transitions. For its calculation, both START_ITEM and END_ITEM were excluded because they are administrative actions. Specifically, let $g_{i}$ denote the decision-stage index associated with action $a_{i}$ as Table A1 shows. These stage indices describe positions in the task interface and are separate from the nominal action-state labels. Selections of network, fare, ticket-type, trip-number, and purchase confirmation were assigned indices 1 through 5, respectively. The Cancel action was assigned index 0 because it clears the current selections and returns the interface to the initial state. Therefore, BP is defined as

(4) $$\mathrm{BP}\left(a\right)=\frac{\sum_{i=2}^{\left|a\right|}I\left\{g_{i}<g_{i-1}\right\}}{|a|-1},$$

For BP, the denominator includes all adjacent transitions in a. A higher BP indicates a greater proportion of transitions involving a return to the beginning of the task.

Under the TICKET navigation rules, a decrease in the decision-stage index corresponds to a cancellation following a substantive selection. An initial cancellation was not counted as backtracking because no substantive selection had yet been made. A cancellation following a substantive selection was counted even when the student subsequently ended the item. For consecutive cancellations, only the first was counted. Changes between options within the same decision stage were not counted as backtracking.

Let $a_{i}$ and $a_{j}$ denote the action sequences of students *i* and *j*, and let ${\mathrm{LCS}}_{ij}$ denote the LCS of their action sequences. Pairwise action-path similarity was defined as

(5) $$s_{ij}={\left(\frac{|{\mathrm{LCS}}_{ij}|}{|a_{i}|}\;\frac{|{\mathrm{LCS}}_{ij}|}{|a_{j}|}\right)}^{1/2}=\frac{|{\mathrm{LCS}}_{ij}|}{\sqrt{|a_{i}\left|\;\right|a_{j}|}}.$$

The geometric normalization reduces the influence of sequence length; a larger $s_{ij}$ means two students share a longer ordered action backbone.

Behavioral and timing differences between resilient and non-resilient students were evaluated using two-sided Wilcoxon signed-rank tests to preserve the one-to-one matched-pair structure. Effect sizes were summarized using matched-pairs rank-biserial correlations, with 95% confidence intervals obtained from 5000 bootstrap resamples of complete matched pairs. The comparisons covered sequence length, BP, first-response time, mean action interval, and total task duration. As a sensitivity analysis, the same paired comparisons were repeated among the 53 matched pairs in which both students answered CP038Q02 correctly.

### 3.7. Behavioral Profile Identification and Association Analysis

A weighted undirected graph G=(V,E,s) was constructed with students as nodes and $s_{ij}$ as edge weights. To retain substantively similar paths and reduce weak-similarity noise, the threshold graph kept edges satisfying $E_{\kappa }=\left\{\left(i,j\right):s_{ij}\geq \kappa \right\}$ with κ=0.5. This threshold was an exploratory choice on the normalized similarity scale and was not optimized against an external criterion. Behavioral profiling then used a cluster-editing logic (Bansal et al., 2004; Böcker & Baumbach, 2013). Let $x_{ij}=1$ if the edge between students *i* and *j* is retained and $x_{ij}=0$ otherwise. The objective was $\max\sum_{i<j}s_{ij}x_{ij}$ subject to constraints that remove induced three-node paths,

(6) $$\begin{array}{cccccc} x_{ij}+x_{jh}-x_{ih} & \leq 1,\; & x_{ij}-x_{jh}+x_{ih} & \leq 1,\; & -x_{ij}+x_{jh}+x_{ih} & \leq 1, \end{array}$$

for all i<j<h, with $x_{ij}\in \left\{0,1\right\}$ and non-edges fixed at zero. The edited graph becomes a disjoint union of cliques, each interpreted as a behavioral profile whose members are mutually similar under the threshold criterion. Because the threshold was fixed rather than optimized against an external criterion, the resulting profile solution is interpreted as specific to the selected sample and similarity threshold.

For computational efficiency, identical action sequences were collapsed into exact-sequence supernodes. Backbone optimization used the 80 largest supernodes, representing 353 students, and the remaining supernodes were subsequently assigned using the fixed all-pairs similarity rule. Final profile membership was then mapped back to individual students.

Before any distribution-level analysis, the study tested whether behavioral profile membership was associated with resilience status. Because several long-tail profiles contained very few students, the smallest profiles with fewer than five students were grouped into a small/other category for stable reporting. The resulting profile-by-resilience table was summarized with Pearson’s chi-square statistic, Cramér’s *V*, a pair-preserving permutation *p* value, and a Pearson-residual heatmap, where

(7) $$V=\sqrt{\frac{\chi^{2}}{N\min(R-1,C-1)}},$$

with *R* and *C* the numbers of rows and columns. Statistical significance was evaluated using 5000 permutations in which resilience labels were retained or exchanged only within each matched pair, while behavioral-profile membership remained fixed. Pearson residuals were used to describe the profile–resilience combinations contributing to the overall association.

### 3.8. Supplementary Coupling Analysis

As a supplementary check on whether the profile–resilience alignment exceeds chance, optimal transport (OT) was used to compare two distributions: a distribution over behavioral profiles and a distribution over resilience status. Let $p=(p_{1},\ldots ,p_{K})$ with $p_{k}=n_{k}/N$ denote the profile distribution and $q=(q_{1},q_{2})$ the resilience-status distribution. The cost of matching profile *k* to resilience group *m* combined sequence dissimilarity and behavioral-feature difference,

(8) $$C_{km}=\alpha \;d_{\mathrm{seq}}\left(r_{k},r_{m}\right)+\left(1-\alpha \right){∥t_{k}-t_{m}∥}_{2},$$

where $r_{k},r_{m}$ are representative sequences, $d_{\mathrm{seq}}$ is an LCS-based dissimilarity, and $t_{k},t_{m}$ are standardized behavioral-feature vectors. Representative sequences were observed medoid sequences within the corresponding behavioral profile or resilience group. The feature vectors included first-response time, mean action interval, interval variance, and BP, and were standardized across the principal matched sample. Interval variance was included only in the supplementary OT–CSI feature representation to capture within-sequence variability in action timing; it was not treated as a separate outcome in the paired behavioral comparisons. The entropy-regularized OT plan Γ was estimated with a Sinkhorn-type solution (Cuturi, 2013; Peyré & Cuturi, 2019; Sinkhorn, 1967; Villani, 2009), minimizing $\sum_{k,m}\Gamma_{km}C_{km}+\varepsilon \sum_{k,m}\Gamma_{km}\left(\log\Gamma_{km}-1\right)$ subject to Γ1=p and $\Gamma^{⊤}1=q$.

To judge whether the observed alignment was stronger than chance, resilience labels were permuted within matched pairs while the action sequences and profile structure were held fixed. The *Coupling Strength Index* (CSI) is the relative reduction in observed transport cost compared with the expected cost under random permutation,

(9) $$\mathrm{CSI}=\frac{E\left({\mathrm{Cost}}_{\mathrm{rand}}\right)-{\mathrm{Cost}}_{\mathrm{obs}}}{E({\mathrm{Cost}}_{\mathrm{rand}})}.$$

Here *coupling* refers only to statistical alignment between the two distributions. This supplementary procedure is referred to hereafter as the OT–CSI analysis. A positive CSI means the observed transport cost is lower than the expected random cost; it is a relative cost-reduction index, not a causal effect size, and does not show that a particular action path causes resilience. The empirical permutation value used add-one smoothing, $p_{\mathrm{perm}}=\left(1+\#\left\{b:{\mathrm{Cost}}_{\mathrm{rand}}^{\left(b\right)}\leq {\mathrm{Cost}}_{\mathrm{obs}}\right\}\right)/\left(B_{\mathrm{perm}}+1\right)$. The supplementary analysis used α=0.5, ε=0.03, and 5000 independent within-pair label permutations. In each permutation, resilience labels were either retained or exchanged within each matched pair, while profile membership remained fixed. The smallest reportable permutation value was therefore 1/5001<0.001.

The OT–CSI analysis was treated as a supplementary distribution-level summary of the association between behavioral structure and resilience status and was interpreted together with the profile–resilience association analysis.

As a completed-dataset robustness analysis, the five paired behavioral comparisons, behavioral-profile identification and profile–resilience association analysis, and supplementary OT–CSI analysis were repeated separately in the matched sample obtained from each of the 20 completed datasets using the same analytical specifications as in the principal analysis.

All analyses were conducted in Python 3.13.7 using NumPy 2.3.5, pandas 3.0.1, SciPy 1.16.3, and pyreadstat 1.3.3. The graph-clustering mixed-integer linear programs were solved with Gurobi Optimizer 12.0.2 (gurobipy 12.0.2) under Python 3.11.4. Figures were generated in R 4.5.0.

## 4. Results

### 4.1. Covariate Balance

The final matched sample contained 220 pairs (N=440), selected from 708 eligible candidates in the randomly selected seventh completed dataset. Each pair included one resilient and one non-resilient student. The behavioral comparison was made between disadvantaged students whose observed background characteristics were made more comparable before their process data were analyzed. The subsequent behavioral comparisons therefore concern the principal matched sample. Diagnostic summaries across all 20 completed datasets are reported in the Appendix B.

**Figure 3:**
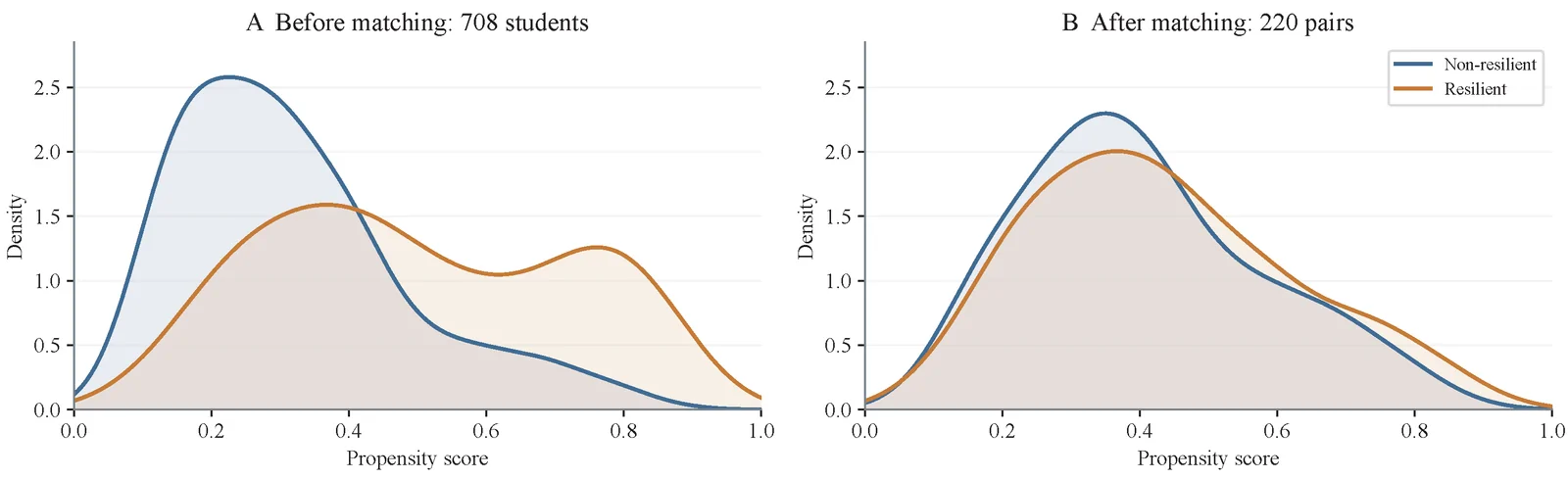
Propensity-score distributions before (**A**) and after (**B**) pooled matching in the seventh completed dataset.

Figure 3 shows that propensity-score overlap improved after matching. This pattern indicates improved comparability on observed covariates. Table 2 reports the SMD diagnostics. The largest pre-matching differences were observed for country and MMINS, and both were substantially reduced after matching. Including country, the maximum absolute term-level SMD decreased from 0.654 before matching to 0.137 after matching. Five of the seven selected covariates had post-matching absolute SMDs below 0.1. Residual imbalance remained for IMMIG (|SMD|=0.108) and MMINS (|SMD|=0.137), while the country term had |SMD|=0.114. Across the 20 completed datasets, the complete summaries of balance diagnostics are reported in the Appendix B.

### 4.2. Behavioral Profiles in the TICKET Task

**Figure 4:**
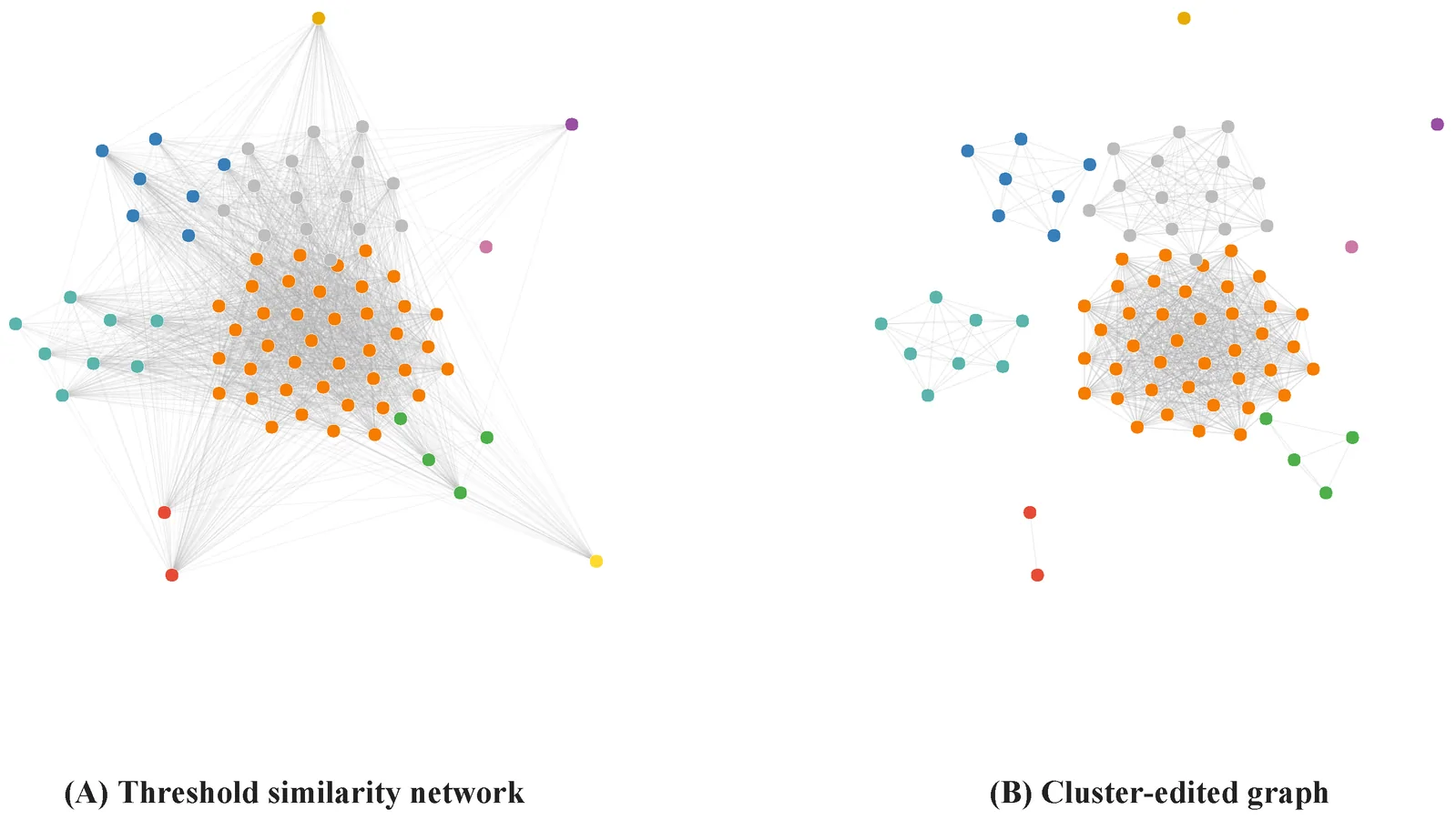
Threshold similarity network and cluster-edited graph at κ=0.5. Nodes represent the 80 largest exact-sequence supernodes used in the backbone optimization, and colors indicate their final behavioral-profile membership. Panel (**A**) shows the threshold network and Panel (**B**) the cluster-edited graph.

LCS similarity and cluster edge deletion identified K=14 behavioral profiles. Figure 4 shows the threshold network and the edited graph. The TICKET responses were neither completely homogeneous nor completely idiosyncratic: several students shared interpretable routes, while a small number of long-tail paths remained individualized. The network visualization contains the 80 largest exact-sequence supernodes used in the backbone optimization, representing 353 students. The remaining supernodes were subsequently assigned using the fixed all-pairs rule, and the final profile counts include all 440 students. The resulting K=14 solution is interpreted as specific to the selected sample and similarity threshold.

**Figure 5:**
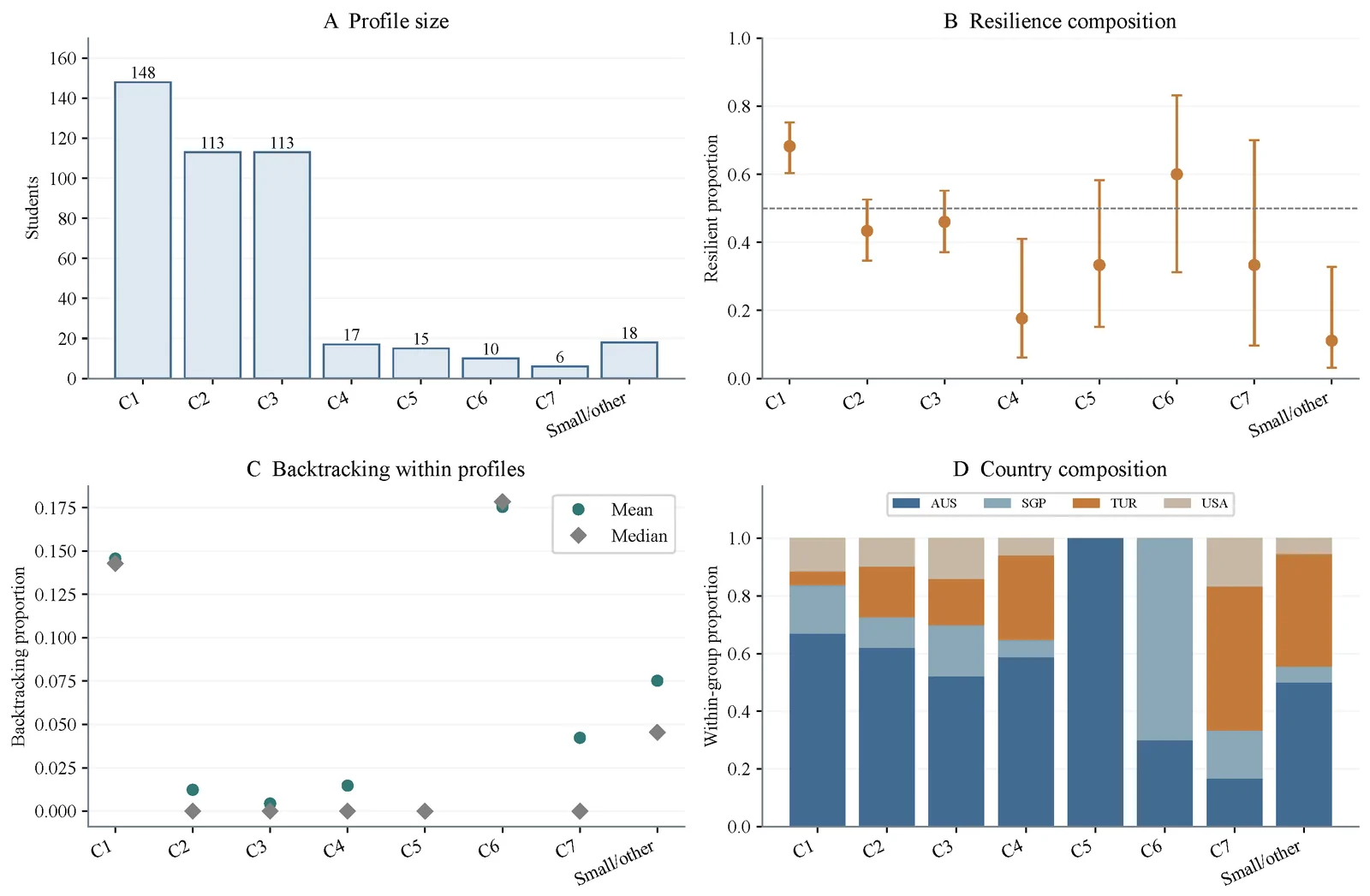
Behavioral-profile summaries in the 440-student matched sample: profile size (**A**), resilience composition (**B**), backtracking proportion (**C**), and country composition (**D**). The dotted line in panel (**B**) indicates the overall proportion of resilient students (50%) in the matched sample.

Figure 5 summarizes profile size and resilience proportion, and Table 3 reports the main profiles. C1 was the largest profile, with 148 students, followed by C2 and C3 with 113 students each. Together, these three profiles accounted for 374 of the 440 students (85.0%). C1 involved repeated ticket choices and cancellation before completion and had a mean backtracking proportion of 0.146. In comparison, C2 and C3 followed more direct routes and had mean backtracking proportions of 0.012 and 0.004, respectively. The observed resilient proportions were 68.2% in C1, 43.4% in C2, and 46.0% in C3. The smaller profiles are retained for completeness but do not carry the main interpretation because their sample sizes are limited. The smaller profiles are interpreted descriptively and are not used to support profile specific inferential claims. In particular, profiles with fewer than five students were combined into the Small/other category for the profile–resilience association analysis.

### 4.3. Association Between Profiles and Resilience Status

**Figure 6:**
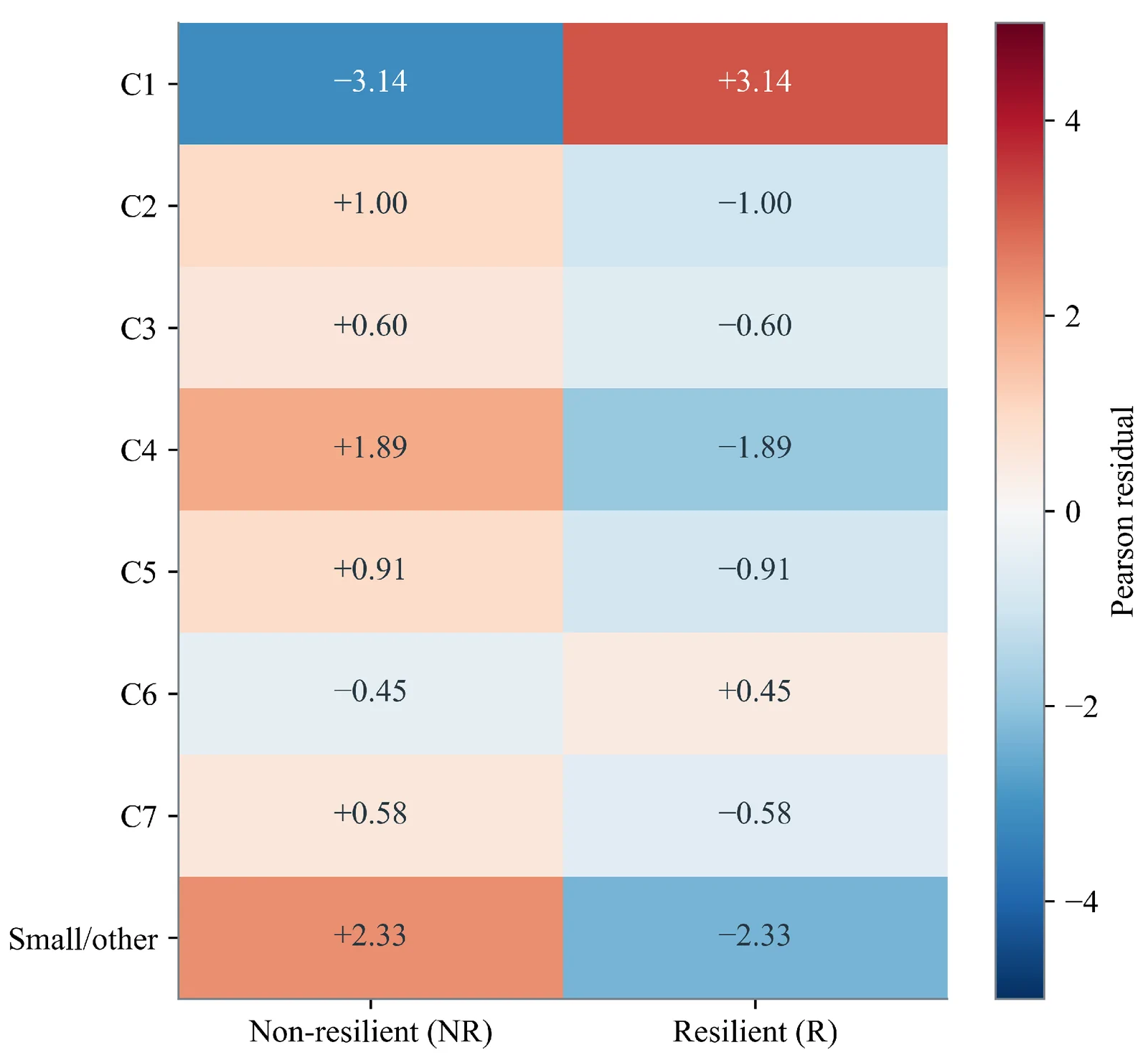
Pearson-residual heatmap for the profile-by-resilience association in the 440-student matched sample.

For the association check, the seven main profiles (C1–C7) were retained as separate rows and the remaining seven small long-tail profiles (each n<5) were combined into a single Small/other group, giving an 8×2 table (Table 4). The table showed a statistically detectable association of moderate strength, $\chi^{2}\left(7\right)=43.151$, permutation p<0.001, Cramér’s V=0.313. Behavioral profiles were thus not independent of resilience status in the matched sample. Figure 6 shows the Pearson residuals: C1 had the largest positive residual for resilient students, whereas C4 and the Small/other category showed negative residuals for resilient students. No separate significance tests were conducted for individual profiles. Statistical inference was based on the single omnibus pair-preserving permutation test, while Pearson residuals were used descriptively to characterize contributions to the overall association.

Sensitivity analyses using κ=0.40, 0.45, 0.50, 0.55, and 0.60 showed that the exact number of profiles was threshold-dependent, ranging from 8 to 27. However, the profile–resilience association remained statistically detectable under pair-preserving permutation at all five thresholds, with Cramér’s *V* ranging from 0.282 to 0.356. Agreement with the reference solution at κ=0.50 was highest at the adjacent thresholds of 0.45 and 0.55. Detailed results are provided in Appendix D Table A9.

### 4.4. Behavioral Differences Between Resilient and Non-Resilient Students

**Figure 7:**
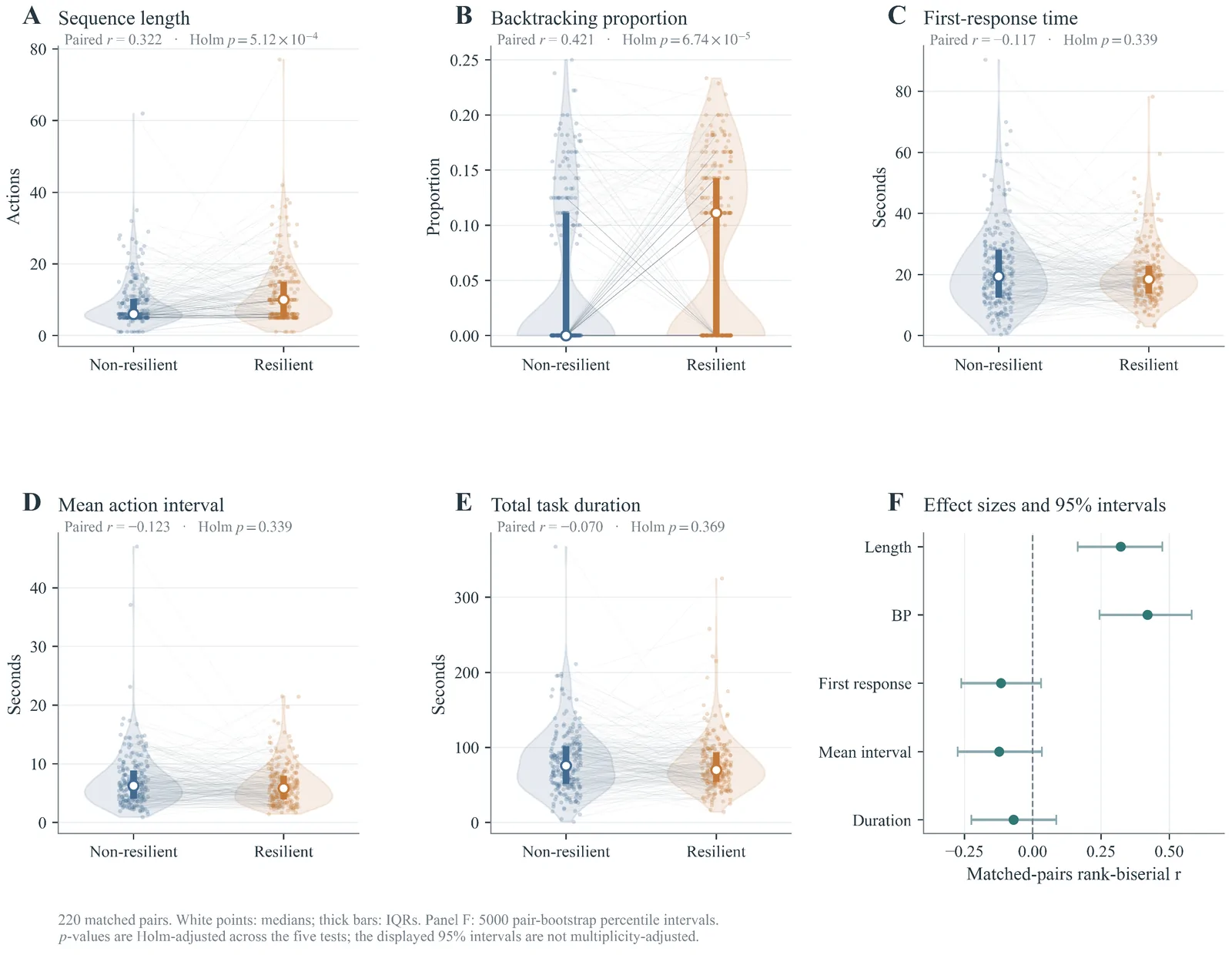
Behavioral distributions and paired effect sizes for 220 matched pairs. Panels (**A**–**E**) show observed values, distribution shapes, pair connections, medians, and interquartile ranges. Panel (**F**) shows matched-pairs rank-biserial correlations and 95% percentile intervals based on 5000 bootstrap resamples of the matched pairs. Two-sided Wilcoxon signed-rank *p* values were adjusted across the five primary comparisons using the Holm procedure.

The central behavioral result concerns how the two resilience-status groups differed in their observed task behavior. Figure 7 compares resilient and non-resilient students on sequence length, first-response time, mean action interval, total task duration, and the backtracking proportion. Table 5 reports the corresponding group medians for the five behavioral measures. Because the analysis preserved the matched-pair design, each feature was tested with a two-sided Wilcoxon signed-rank test and summarized using the matched-pairs rank-biserial correlation.

Resilient students tended to have longer sequences (median =10 vs. 6.000; r=0.322, 95% bootstrap interval [0.165, 0.475], $p_{\mathrm{Holm}}=0.000512$) and higher backtracking proportions (median =0.111 vs. 0.000; r=0.421, 95% bootstrap interval [0.245, 0.582], $p_{\mathrm{Holm}}=0.0000675$). In contrast, first-response time (r=−0.117, $p_{\mathrm{Holm}}=0.339$), mean action interval (r=−0.123, $p_{\mathrm{Holm}}=0.339$), and total task duration (r=−0.070, $p_{\mathrm{Holm}}=0.369$) did not show statistically significant paired differences. Median total task duration was 69.750 s in the resilient group and 75.6 s in the non-resilient group. These statistically significant behavioral differences indicate associations with resilience status.

### 4.5. Supplementary OT–CSI Analysis

The supplementary OT–CSI analysis pointed in the same direction. With α=0.5, ε=0.03, and 5000 within-pair label permutations, the observed transport cost (0.577) was lower than the random-permutation expectation (mean null cost =0.678), giving CSI=0.149 and p<0.001. None of the 5000 permuted costs was at or below the observed cost. The lower observed cost means that the empirical profile distribution aligned with resilience status at lower cost than after pair-preserving random reassignment of resilience labels. This result indicates distribution-level alignment between behavioral profiles and resilience status under the specified cost function and fixed profile assignment. The OT–CSI result therefore provides supplementary evidence consistent with the profile-by-resilience association reported above.

**Figure 8:**
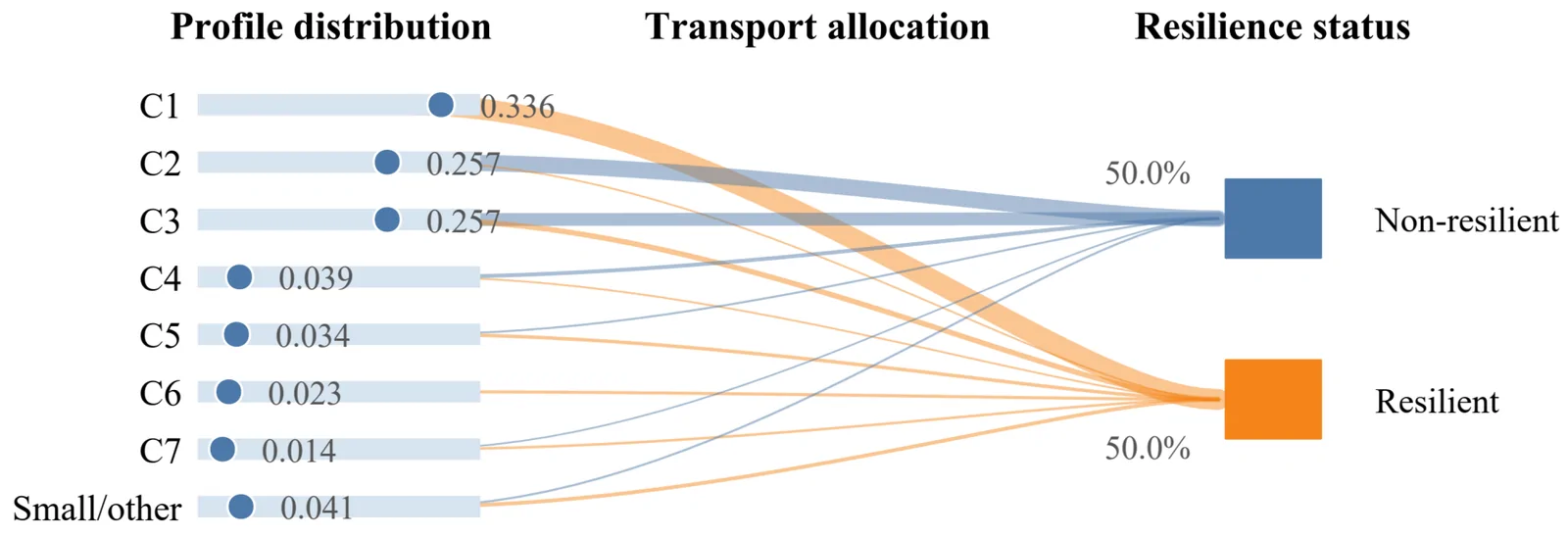
Estimated optimal-transport allocation between behavioral-profile and resilience-status distributions in the principal matched sample. Line widths represent the estimated transport allocation at the distribution level.

Figure 8 summarizes the estimated transport allocation underlying this distribution-level alignment. Transport allocation is constrained by the full coupling problem and depends on both the behavioral cost matrix and the marginal distributions. The line widths therefore represent estimated transport mass between profile and resilience distributions.

To evaluate whether the principal findings depended on the randomly selected seventh completed dataset, the downstream analyses were repeated separately in the matched samples obtained from all 20 completed datasets. The sequence-length and backtracking-proportion effects were in the same direction in all 20 datasets and remained statistically significant after Holm adjustment in all 20. Their median matched-pairs rank-biserial correlations were 0.288 and 0.374, with ranges of 0.228–0.399 and 0.301–0.471, respectively. First-response time and mean action interval retained the same direction across all 20 datasets but were not statistically significant in any dataset, while total task duration was in the same direction in 18 of 20 datasets and was non-significant throughout (Appendix B Table A5).

The behavioral-profile solutions contained 13–17 profiles across the 20 datasets, with a median of 14. The profile–resilience association remained statistically detectable under pair-preserving permutation in all 20 datasets, with Cramér’s *V* ranging from 0.269 to 0.374 (median = 0.330). The supplementary CSI was positive in all 20 datasets, ranging from 0.085 to 0.208 (median = 0.148), and the OT permutation test was statistically significant in 19 of the 20 datasets. These results indicate that the principal behavioral findings and profile–resilience association were not specific to the seventh completed dataset, while the OT–CSI evidence remained broadly consistent across completed-data-specific matched samples (Appendix B Table A6).

## 5. Discussion

This study addressed a central limitation of outcome-based resilience research. Academic resilience is usually identified after the assessment, but process logs make it possible to ask whether resilient students also behave distinctively while working through a task. The findings address the three research questions at complementary levels. For RQ1, action sequences in the matched disadvantaged sample formed interpretable behavioral profiles, although the exact profile resolution depended on the sample and similarity threshold. For RQ2, profile membership was associated with resilience status, and this association remained statistically detectable across the examined thresholds and the matched samples obtained from all 20 completed datasets. For RQ3, the largest profiles differed descriptively in their action routes and mean backtracking proportions. At the group level, resilient students showed longer action sequences and higher backtracking proportions, while the examined timing measures did not show statistically significant paired differences after Holm adjustment. A supplementary coupling analysis corroborated this alignment at the distribution level. Together, these findings extend the examination of outcome-based academic resilience to the organization of behavior within a digital task, while leaving the psychological functions of the observed actions open to further investigation.

### 5.1. Adaptive Persistence as a Possible Interpretation

Previous research has examined academically resilient students in relation to school conditions, self-beliefs, and engagement in learning (Agasisti et al., 2021; K. C. Cheung et al., 2024). Classroom observations and questionnaire studies have also associated academic resilience with instructional participation and school engagement (Padron et al., 1999; Romano et al., 2021). The present study extends this literature to the organization of actions within a specific digital task. In the matched disadvantaged sample, students meeting a mathematics-based resilience criterion differed in their action-sequence characteristics and behavioral-profile membership. This extension concerns the level at which behavior is examined, from broader participation in learning to the ordering of selections, cancellations, and subsequent actions during task performance. It does not establish that school engagement and task-level backtracking reflect the same psychological construct.

One possible interpretation of the observed behavioral pattern is adaptive persistence in a digital problem-solving task. We use the term to describe continued engagement with a task that includes trying alternatives, reconsidering earlier selections, revising previous selections, and continuing toward completion. This interpretation is conceptually consistent with individual-differences work on adaptive and complex problem solving, where responses to uncertainty and changing task states are important aspects of problem-solving behavior (Dörner & Funke, 2017; Eichmann et al., 2019; Greiff et al., 2013; Wüstenberg et al., 2012).

The findings also connect with the process-data literature reviewed in Section 2.2. Eichmann et al. (2020) identified successful problem-solving patterns involving both economical execution and repeated goal-relevant actions. Stadler et al. (2019) found that successful and unsuccessful students using the same exploratory procedure differed in how their actions were organized, although total action counts and working time did not differ significantly. These findings support interpreting the amount of activity together with its sequential context. The present finding of longer sequences among resilient students concerns a different comparison, because group membership was defined by mathematics achievement under socioeconomic disadvantage, not by correctness on the TICKET item. It therefore extends the examination of behavioral heterogeneity to an outcome-based resilience classification without establishing a general relationship between longer paths and successful problem solving.

At the profile level, the representative route for C1 involved repeated ticket choices and cancellations followed by further selections. C1 also contained a higher observed proportion of resilient students than the other two largest profiles. C2 and C3 had more direct representative routes and substantially lower mean backtracking proportions. These patterns suggest that relatively strong mathematics achievement under disadvantage can coexist with revisional task paths. They do not identify a uniformly efficient route characteristic of resilient students. The timing results require a separate interpretation. Previous research has shown that associations between time on task and success depend on task difficulty and individual skill (Goldhammer et al., 2014). Here, first-response time, mean action interval, and total task duration did not show statistically significant paired differences after Holm adjustment. These findings do not establish equivalent timing distributions or demonstrate that the observed revisions were efficient.

Backtracking is also behaviorally ambiguous. A return or cancellation may accompany correction after an unsuccessful choice, but repeated or excessive backtracking may also reflect uncertainty, inefficient search, or difficulty in identifying an appropriate solution path. Thus, a higher backtracking proportion should not be interpreted as inherently beneficial. Its meaning depends on the surrounding action sequence, the task demands, and the eventual task outcome. An important theoretical distinction is between continued interaction with a problem and the productivity of the revisions made during that interaction. A cancellation may allow an earlier choice to be reconsidered, but its potential adaptive value depends on whether subsequent actions better address the task requirements. Repeatedly returning to the same unsuccessful selections could indicate continued effort with limited progress. The present findings therefore motivate examining the conditions under which revision supports productive problem solving. Positive adaptation at the level of mathematics achievement cannot automatically be assigned to every action performed within the digital task.

These observations should remain at the behavioral level. The study did not directly measure persistence, metacognitive monitoring, motivation, or self-regulation. Longer sequences and repeated cancellations therefore cannot be taken as direct evidence of these psychological processes. Adaptive persistence is used here as an interpretive possibility that is consistent with the observed pattern, not as an empirically established mechanism. Moreover, because mathematics achievement contributed to the resilience classification, the observed behavioral differences may also reflect differences in academic competence. The contribution is thus to identify task-level behavioral correlates of outcome-based academic resilience and to specify questions about their psychological functions that require direct measurement and further investigation.

### 5.2. Methodological Contribution

Secondarily, the study contributes a modular process-data workflow that combines LCS similarity, graph-based cluster editing, an explicit profile–resilience association check, and a supplementary coupling index within one exploratory pipeline. Previous research has combined sequence similarity and graph-based clustering to identify common response processes (Ulitzsch et al., 2021), and has used biclustering to examine heterogeneity in TICKET process data (Xu et al., 2024). The present study builds on these approaches by connecting behavioral-profile identification with a matched comparison based on an externally defined academic-resilience classification. Its methodological contribution lies in coordinating sample construction, sequence representation, and statistical evaluation to examine how outcome-defined groups differ in their task behavior.

Each component serves a distinct purpose. PSM reduces observed background differences between the resilience groups before their process outcomes are compared. LCS similarity uses the relative ordering of shared actions to compare response paths, while graph-based profiling summarizes the multiple routes represented in the matched sample. These sequence-based descriptions complement the comparisons of individual behavioral indicators. Sequence length and BP quantify the amount of recorded activity and the proportion of backtracking transitions, but do not fully describe how selections and cancellations are combined within a response path. Behavioral profiles provide this additional context. The combination therefore connects group-level differences in summary indicators with the action patterns underlying those differences.

This separation of analytical roles is particularly useful for studying academic resilience. Resilience status was defined from socioeconomic background and mathematics achievement before the process-data analysis. The behavioral profiles were subsequently constructed from action sequences, without using resilience labels to define sequence similarities or determine profile membership. The association analysis then examined whether these profiles were distributed differently between the two resilience groups. This design keeps the definition of academic resilience separate from the behavioral patterns examined as its potential correlates. Pair-preserving permutation procedures evaluate the profile–resilience association while retaining the matched-pair structure. The supplementary OT–CSI analysis adds a distribution-level comparison that incorporates sequence dissimilarities and behavioral-feature distances alongside profile frequencies. It provides a complementary description of alignment without assigning psychological meaning to individual actions.

The sensitivity analyses further distinguish the robustness of the overall association from the stability of a particular profile solution. Varying the LCS threshold examines how the findings depend on the similarity requirement used to construct profiles. Repeating the downstream analyses in the matched samples obtained from all 20 completed datasets examines sensitivity to the background-data completion and the resulting selection and pairing of students. These checks allow changes in profile resolution and effect magnitude to be reported alongside the consistency of the main findings. They assess robustness within the available data and do not constitute independent replication. The methodological contribution is therefore an exploratory framework for describing relations between process structure and an externally defined resilience classification, while the strength and reproducibility of those relations require further evaluation.

### 5.3. Implications

The present findings suggest that outcome-based classifications of academic resilience can be supplemented by information about how students interact with a digital task. In this task, resilience status was associated with longer and more cancellation-involving action paths. This finding reinforces an important point for process-data interpretation. A cancellation or return is not automatically a sign of poor performance. Depending on its position in the sequence, it may accompany correction or continued search, although the same behavior can also indicate uncertainty or inefficiency. The relevant unit of interpretation is therefore the action in its sequential context. Its frequency alone provides limited information about whether the student reconsidered an earlier choice productively or continued an unsuccessful search.

For assessment, the practical upshot is that process data and scores answer different questions. A score reports whether a student reached a successful outcome; a path provides information about the sequence of actions through which that outcome was approached. An assessment report could therefore combine accuracy with task-specific descriptions of action sequences, including whether students followed direct routes or reconsidered selections after cancellation. Such descriptions can distinguish response processes that an item score alone cannot differentiate. However, the present associations provide no basis for automatically rewarding backtracking, penalizing longer paths, or treating rapid completion as evidence of better problem solving. Any use of these indicators in scoring would require separate evidence that the indicators support the intended assessment interpretation.

Process traces alone, however, do not identify the psychological processes producing those actions. The behavioral profiles are therefore most useful when interpreted alongside achievement, questionnaire measures, and information about the learning context. They should not be used as standalone diagnoses of individual students. In a formative setting, repeated cancellation could prompt closer examination of which selections were reconsidered and invite students to explain the reasons for those changes. This is a potential use of process information to guide further inquiry, whose educational value would need to be evaluated. The present study did not test feedback or instructional interventions and does not show that encouraging more revisions would improve achievement or academic resilience.

For future research, the findings suggest examining when and under what task conditions revision contributes to successful problem solving. Studies involving multiple tasks could investigate whether the association between resilience status and revisional behavior changes with task difficulty, decision structure, and opportunities for correction. Examining the actions following a cancellation could help distinguish revisions that more closely satisfy task requirements from repeated unsuccessful selections. Combining these sequence-level observations with direct measures of persistence, metacognitive monitoring, motivation, and self-regulation would allow the proposed psychological explanations to be tested. Independent studies across tasks and populations would also help establish which behavioral associations are specific to TICKET and which recur under different assessment conditions.

### 5.4. Limitations and Future Work

A number of limitations bound these conclusions. First, the evidence is based on a single PISA 2012 TICKET item. A behavioral pattern observed in one interactive task cannot establish that the same pattern will appear in other digital problem-solving tasks. The four-country pooled sample likewise does not establish replication across countries. Replication would require the association to recur in independent samples or tasks under comparable analytical procedures, whereas broader generalization would require convergent evidence across different tasks, countries, age groups, and assessment contexts. The present findings should therefore be interpreted as task- and sample-specific evidence. In addition, the TICKET task was administered in PISA 2012 and reflects an earlier generation of computer-based assessment; its interface and interaction structure may therefore not fully represent contemporary digital problem-solving environments.

Second, the final matched sample contained 220 pairs (N=440). Although this sample supports the primary paired comparisons, it is relatively limited for a 14-profile solution, particularly for the smaller behavioral profiles. Accordingly, estimates and interpretations involving sparse profiles should be treated cautiously.

Third, PSM improves comparability only on observed covariates. Residual imbalance remained after matching for IMMIG (|SMD|=0.108), MMINS (|SMD|=0.137), and country (|SMD|=0.114). Consequently, some of the observed behavioral differences may still partly reflect residual differences in immigrant background, mathematics instructional time, or country composition. This remaining imbalance, together with potentially relevant unmeasured characteristics, limits the extent to which the observed behavioral contrasts can be attributed specifically to resilience status. In addition, unobserved characteristics, including motivational, instructional, or family-related factors, may be associated with both resilience status and task behavior. The matched design therefore does not support a causal interpretation.

Fourth, the transformation of the raw log files into analytical actions required researcher-defined coding decisions. Different aggregation or coding rules may alter the resulting action sequences and profile structure. The backtracking proportion is also dependent on the researcher-specified ordering of task stages. Although the numerical ordering used here followed the structure of the TICKET task, alternative representations of the task states could lead to different backtracking values. Future studies should examine the robustness of this indicator under alternative coding schemes.

Fifth, the LCS-based clustering used a fixed similarity threshold of κ=0.5. The resulting profile solution is therefore conditional on both the selected sample and this analytical threshold. Alternative thresholds, coding schemes, and clustering specifications should be examined in independent data before the identified profiles are regarded as stable behavioral categories.

Sixth, adaptive persistence, metacognitive monitoring, motivation, and self-regulation were not directly measured. Consequently, these constructs cannot be inferred from backtracking or sequence length alone. Future work should combine process traces with direct questionnaire, experimental, or other measurement evidence for these constructs if the adaptive-persistence interpretation is to be tested more directly.

Seventh, the treatment of PISA achievement and sampling limits the scope of inference. A mathematics plausible value was used as part of the resilience classification, while the full plausible-value inference procedure was not propagated through the sequence analyses. The matching and process analyses also did not incorporate the full PISA complex-sampling and replicate-weight variance estimation framework. The present results should therefore be interpreted as associations within the analyzed matched sample and not as nationally representative population estimates.

Finally, uncertainty associated with the completed background-data sets was not formally pooled across the process-data analyses. Although the paired behavioral comparisons, behavioral-profile analyses, and supplementary OT–CSI analysis were repeated across all 20 completed-data-specific matched samples and showed broadly stable conclusions, the detailed profile descriptions and visualizations were based on the randomly selected seventh completed dataset. Future work could develop procedures that jointly propagate missing-data, matching, and sequence-analysis uncertainty.

Future studies should therefore prioritize independent replication across tasks and populations, alternative action codings and similarity thresholds, and the integration of direct psychological measures with process traces. Timing information could also be incorporated more directly into sequence similarity measures to examine whether action order and action timing provide complementary evidence about problem-solving behavior.

## 6. Conclusions

Using PISA 2012 TICKET-task logs, this study examined academic resilience through action-path similarity in a matched sample of disadvantaged students. The study extends the examination of outcome-based academic resilience to the organization of behavior within a specific digital task. Students classified as resilient showed longer action sequences and higher backtracking proportions, and behavioral-profile membership was associated with resilience status. The principal behavioral contrasts were consistent across the completed-data-specific matched samples, and the profile–resilience association was retained across the examined similarity thresholds. The timing measures did not show statistically significant paired differences after Holm adjustment. Together, these findings show that relatively strong mathematics achievement under socioeconomic disadvantage can coexist with revisional task paths. The findings therefore motivate distinguishing continued interaction with a task from the productivity of individual revisions when interpreting behavioral correlates of academic resilience.

Methodologically, the study illustrates how matching, whole-sequence similarity, graph-based profiling, and pair-preserving inference can be combined to examine behavioral correlates of an externally defined achievement-based classification. Sensitivity analyses assess the dependence of these associations on analytical choices, while OT–CSI provides a supplementary distribution-level perspective. The contribution remains behavioral and associational. The single PISA 2012 item, pooled four-country matched sample, residual covariate imbalance, possible unmeasured confounding, and absence of direct psychological measures constrain the interpretation and generalizability of the findings. Adaptive persistence remains one possible explanation of the recorded patterns, alongside uncertainty and inefficient search. Independent studies incorporating multiple tasks and direct psychological measures are needed to evaluate these explanations and their applicability beyond the present assessment context. Process logs thus provide a useful complement to achievement scores by showing how students work through complex digital tasks, not only how they score at the end.

## Figures and Tables

**Table 1:** Sample construction and country composition of the principal matched sample.

Country	Initial Eligible Sample	Complete Data for Classification	Eligible Disadvantaged Students	Resilient Candidates	Matched Resilient	Comparison Candidates	Matched Comparison	Final N
Australia	14,481	14,110	3574	135	130	216	136	266
Singapore	5546	5519	1380	85	38	29	29	67
Turkey	4848	4806	1232	29	29	131	31	60
United States	4978	4915	1243	25	23	58	24	47
Total	29,853	29,350	7429	274	220	434	220	440

Note. The first three columns describe the national source samples before restriction to TICKET participants. The 708 matching candidates were obtained after the missing-data procedure. Pooled matching allowed cross-country pairs. The seventh completed dataset yielded 220 matched pairs (440 distinct students) and was used for the principal behavioral analyses.

**Table 2:** Covariate balance before and after pooled matching in the seventh completed dataset.

Covariate	Pre |SMD|	Post |SMD|	Balance
ST04Q01	0.222	0.018	Good
IMMIG	0.228	0.108	Mild
FAMSTRUC	0.099	0.080	Good
TEACHSUP	0.191	0.031	Good
STUDREL	0.095	0.037	Good
MTSUP	0.017	0.032	Good
MMINS	0.615	0.137	Mild
CNT	0.654	0.114	Mild

Note. Good denotes |SMD|<0.1 and Mild denotes 0.1≤|SMD|<0.15.

**Table 3:** Main behavioral profiles in the 440-student matched sample. Representative routes summarize substantive task actions.

Profile	*n*	Resil. Prop.	Representative Route	Backtrack. Prop.	Interpretation
C1	148	0.682	City subway–concession–individual/trip 4–Cancel; city subway–concession–daily–Cancel; then city subway–concession–individual/trip 4–Buy	0.146	Largest profile; repeated ticket choices and cancellation before completion
C2	113	0.434	City subway–concession–daily–Buy	0.012	Mainstream short route with little backtracking
C3	113	0.460	City subway–concession–individual–trip 4–Buy	0.004	Direct individual-ticket route with little backtracking
C4	17	0.176	Country trains–concession–daily–Buy	0.015	Short route with a low observed resilient proportion
C5	15	0.333	No substantive task action	0.000	No substantive recorded action before termination
C6	10	0.600	Repeated fare and ticket selections with Cancel cycles, then Buy	0.175	Long revisional route with the highest mean backtracking proportion; interpreted cautiously because of the small profile size
C7	6	0.333	Country trains–concession–individual–trip 2–Buy	0.042	Small direct-route profile; not used as major evidence
Small/other	18	0.111	Mixed long-tail routes	—	Seven sparse profiles, each with n<5; reported for completeness

**Table 4:** Aggregated profile-by-resilience contingency table (N=440).

Profile Group	Resilient *n* (Row %)	Non-Resilient *n* (Row %)	Row Total
C1	101 (68.2)	47 (31.8)	148
C2	49 (43.4)	64 (56.6)	113
C3	52 (46.0)	61 (54.0)	113
C4	3 (17.6)	14 (82.4)	17
C5	5 (33.3)	10 (66.7)	15
C6	6 (60.0)	4 (40.0)	10
C7	2 (33.3)	4 (66.7)	6
Small/other	2 (11.1)	16 (88.9)	18
Total	220 (50.0)	220 (50.0)	440

Note. Row percentages are within-profile shares. Small/other combines seven profiles with fewer than five students each.

**Table 5:** Paired behavioral comparisons.

Outcome	R Median	NR Median
Sequence length	10.000	6.000
BP	0.111	0.000
First-response time	18.500	19.400
Mean action interval	5.818	6.285
Total duration	69.750	75.600

Note. Time outcomes are measured in seconds. R denotes resilient and NR non-resilient.

**Figure A2:**
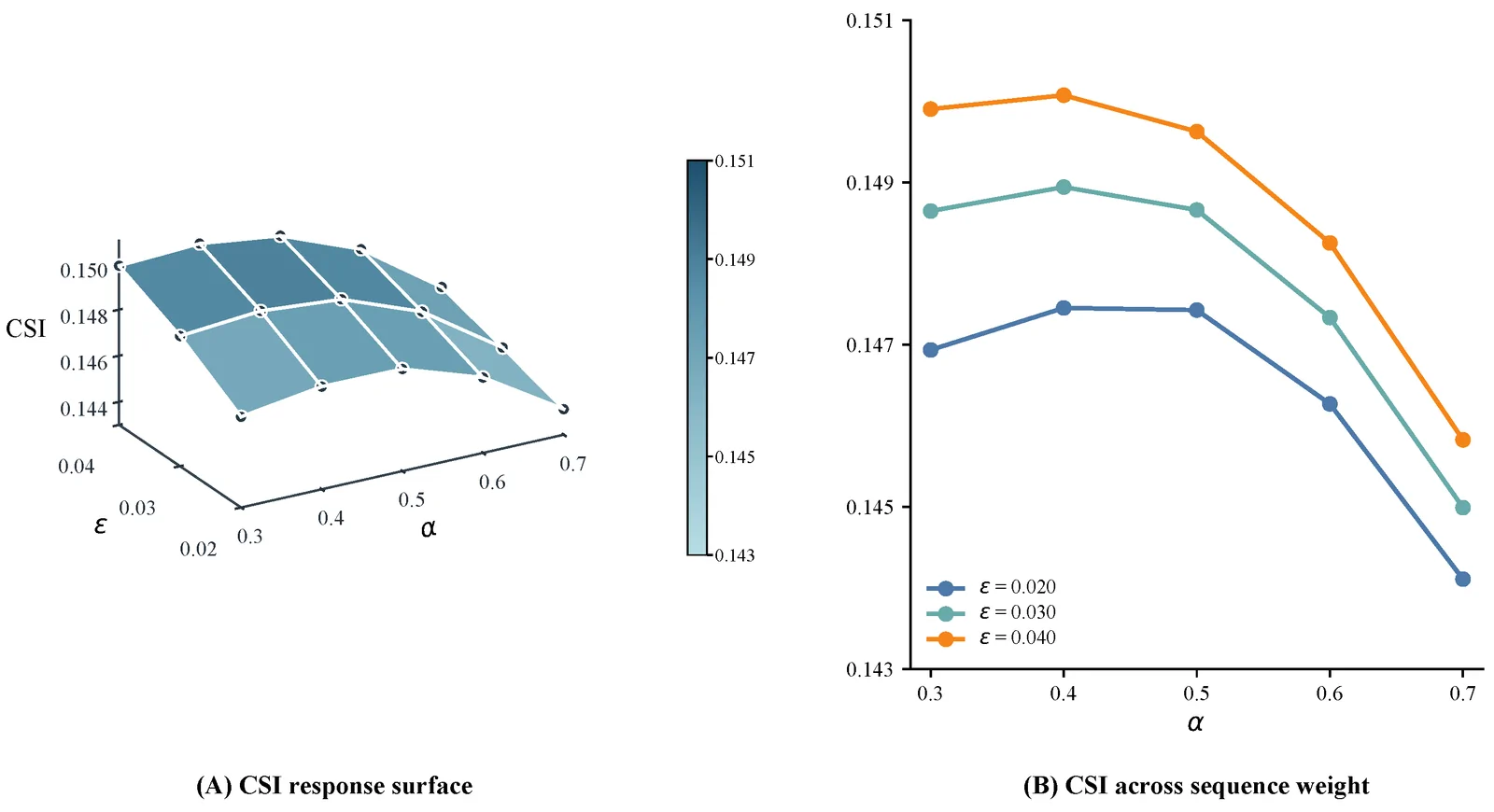
Fixed-sample sensitivity of CSI across 15 combinations of α and ε in the principal matched sample.

**Figure A3:**
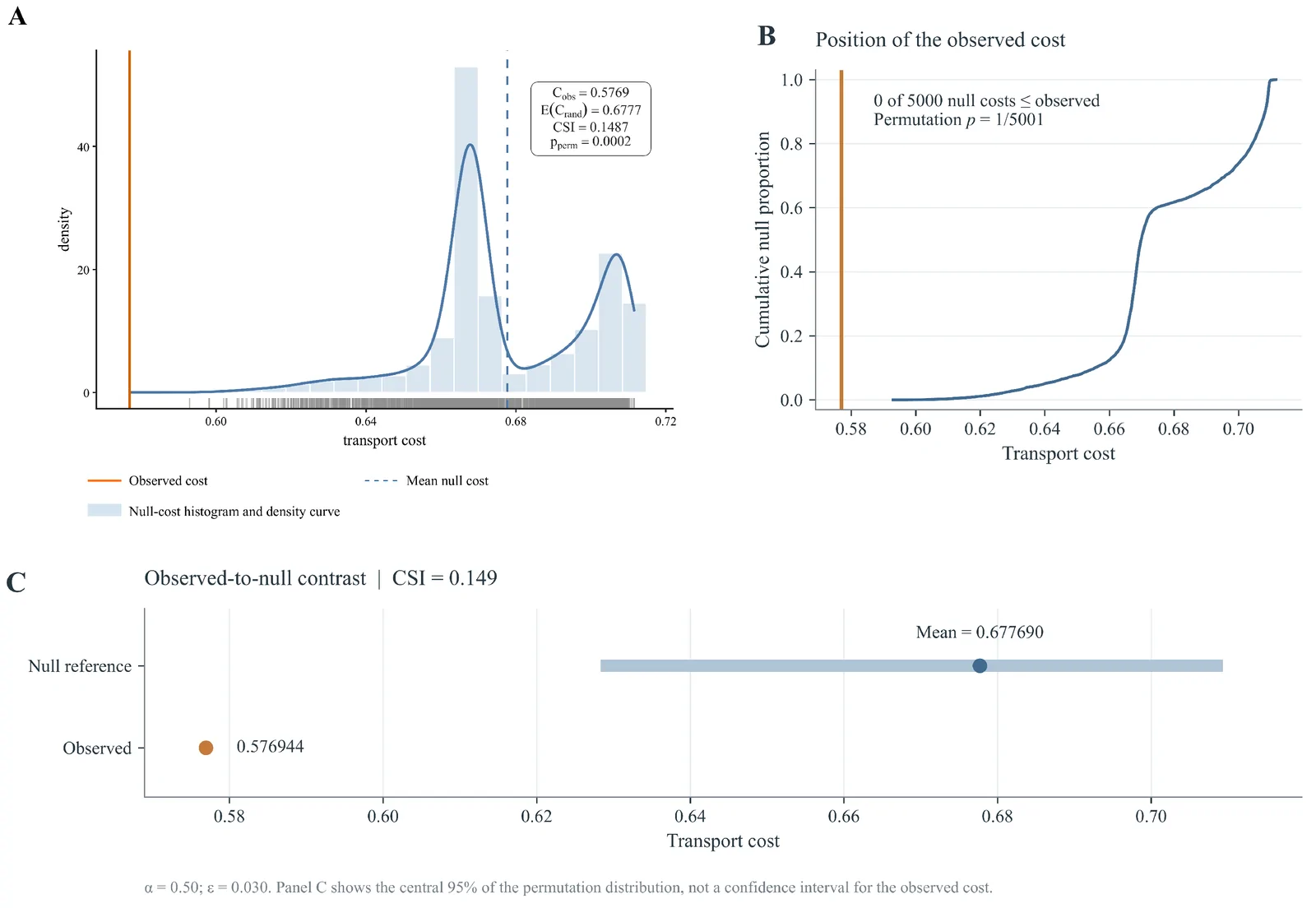
Pair-preserving OT–CSI analysis using 5000 within-pair label permutations. (**A**) Null distribution of transport costs. The light-blue histogram and blue density curve represent the permutation null distribution, the solid orange line indicates the observed cost, and the dashed blue line indicates the mean null cost. (**B**) Cumulative proportion of null transport costs (blue curve), with the solid orange line indicating the observed cost. (**C**) Comparison of the observed transport cost (orange point) and the mean null cost (blue point). The light-blue horizontal interval represents the central 95% of the permutation null distribution, rather than a confidence interval for the observed cost.

## Data Availability

The data used in this study were obtained from the publicly available PISA 2012 database released by the OECD. The processed analytical data and code can be made available by the corresponding author upon reasonable request, subject to the data-use conditions of the original source.

## Appendix A. TICKET Task Interface and Action Codes

**Table A1:** Complete set of action-state labels used to represent the recorded interface states and operations in the TICKET task, organized by decision stage.

Stage	State	Meaning
1	country_trains	Select the country-train network
1	city_subway	Select the city-subway network
2	full_fare	Select the full fare
2	concession	Select the concession fare
3	daily	Select a daily ticket
3	individual	Select individual trips
4	trip_1	Select one trip
4	trip_2	Select two trips
4	trip_3	Select three trips
4	trip_4	Select four trips
4	trip_5	Select five trips
5	Buy	Confirm the purchase
0	Cancel	Clear selections and return to the initial screen
NA	START_ITEM	Administrative start marker
NA	END_ITEM	Administrative termination marker

Note. The labels correspond to the fixed observable interface states and operations recorded for CP038Q02. They are descriptive representations of the log states and are not researcher-defined behavioral categories.

## Appendix B. Covariate Balance, Collinearity, and Robustness Across Completed Datasets

Covariate balance and collinearity were evaluated separately in all 20 completed datasets. Table A2 summarizes term-level absolute SMDs before and after matching, Table A3 summarizes the corresponding pre-matching collinearity diagnostics, and Table A4 reports the number of matched pairs and the maximum post-matching absolute SMD in each completed dataset. The reported ranges are observed minimum–maximum ranges across datasets and are descriptive summaries rather than confidence intervals. Table A5 and Table A6 extend these diagnostics to the downstream analyses by summarizing the robustness of the paired behavioral comparisons, behavioral-profile solutions and profile–resilience associations, and the supplementary OT–CSI results across the 20 completed-data-specific matched samples.

**Table A2:** Covariate balance across the 20 completed datasets under pooled matching.

Term	Before Matching: Mean [min, max]	After Matching: Mean [min, max]
ST04Q01	0.222 [0.222, 0.222]	0.027 [0.009, 0.075]
IMMIG	0.228 [0.228, 0.228]	0.067 [0.015, 0.131]
FAMSTRUC	0.099 [0.099, 0.099]	0.077 [0.012, 0.138]
TEACHSUP	0.213 [0.138, 0.268]	0.071 [0.000, 0.165]
STUDREL	0.115 [0.014, 0.206]	0.081 [0.016, 0.159]
MTSUP	0.030 [0.002, 0.100]	0.049 [0.004, 0.097]
MMINS	0.536 [0.424, 0.615]	0.085 [0.035, 0.170]
CNT	0.654 [0.654, 0.654]	0.113 [0.066, 0.176]

**Table A3:** Collinearity diagnostics across the 20 completed datasets before matching.

Term	Df	GVIF: Mean [min, max]	${\mathrm{GVIF}}^{1/(2\mathrm{Df})}$: Mean [min, max]
ST04Q01	1	1.023 [1.016, 1.034]	1.011 [1.008, 1.017]
IMMIG	2	1.048 [1.027, 1.070]	1.012 [1.007, 1.017]
FAMSTRUC	2	1.044 [1.023, 1.068]	1.011 [1.006, 1.017]
TEACHSUP	1	1.478 [1.366, 1.551]	1.215 [1.169, 1.246]
STUDREL	1	1.290 [1.225, 1.337]	1.136 [1.107, 1.156]
MTSUP	1	1.533 [1.455, 1.630]	1.238 [1.206, 1.277]
MMINS	1	1.034 [1.021, 1.050]	1.017 [1.010, 1.025]

**Table A4:** Matching diagnostics across the 20 completed datasets.

Dataset	Matched Pairs	Maximum Post-Match |SMD|
1	214	0.170
2	219	0.145
3	217	0.131
4	213	0.091
5	214	0.163
6	219	0.105
7	220	0.137
8	220	0.138
9	219	0.105
10	213	0.176
11	211	0.119
12	222	0.124
13	216	0.136
14	216	0.141
15	215	0.103
16	211	0.159
17	210	0.138
18	220	0.102
19	218	0.150
20	220	0.135
Mean	216.35	0.133
Minimum	210	0.091
Maximum	222	0.176

Note. Maximum post-match |SMD| was calculated across the seven matching covariates and country. Matching and balance assessment were conducted separately in each completed dataset.

**Table A5:** Robustness of paired behavioral comparisons across the 20 completed datasets.

Outcome	Dataset 7 Paired *r*	Median Paired *r* Across 20 Datasets	Range of Paired *r*	Same Direction as Dataset 7, n/20	Holm-Adjusted p<0.05, n/20
Sequence length	0.322	0.288	[0.228, 0.399]	20/20	20/20
Backtracking proportion	0.421	0.374	[0.301, 0.471]	20/20	20/20
First-response time	−0.117	−0.055	[−0.131, −0.012]	20/20	0/20
Mean action interval	−0.123	−0.132	[−0.185, −0.039]	20/20	0/20
Total task duration	−0.070	−0.072	[−0.138, 0.013]	18/20	0/20

**Table A6:** Robustness of behavioral-profile and OT–CSI results across the 20 completed datasets.

Panel A. Behavioral-profile solutions and profile–resilience associations						
**Dataset**	**Matched Pairs**	**No. of Profiles K**	**Largest Profile (%)**	** $\chi^{2}$ **	**Cramér’s V**	**Permutation p**
1	214	15	33.9	49.646	0.341	<0.001
2	219	14	33.8	46.967	0.327	<0.001
3	217	13	33.4	47.807	0.332	<0.001
4	213	15	33.6	41.774	0.313	<0.001
5	214	13	33.4	55.126	0.359	<0.001
6	219	13	42.9	43.773	0.316	<0.001
7	220	14	33.6	43.151	0.313	<0.001
8	220	13	33.6	45.982	0.323	<0.001
9	219	15	33.6	52.517	0.346	<0.001
10	213	17	41.5	59.461	0.374	<0.001
11	211	13	33.2	47.664	0.336	<0.001
12	222	13	44.6	49.926	0.335	<0.001
13	216	13	31.9	53.084	0.351	<0.001
14	216	13	35.4	40.605	0.307	<0.001
15	215	14	34.7	31.105	0.269	<0.001
16	211	14	32.9	38.867	0.303	<0.001
17	210	14	33.1	39.446	0.306	<0.001
18	220	15	32.7	45.028	0.320	<0.001
19	218	14	43.6	60.619	0.373	<0.001
20	220	14	42.5	54.684	0.353	<0.001
Median	216.5	14	33.6	47.316	0.330	–
Minimum	210	13	31.9	31.105	0.269	–
Maximum	222	17	44.6	60.619	0.374	–
Panel B. Supplementary OT–CSI results						
**Dataset**	**Observed OT Cost**	**Mean Null Cost**	**CSI**	**Permutation p**		
1	0.572	0.660	0.133	<0.001		
2	0.590	0.690	0.145	<0.001		
3	0.583	0.681	0.143	<0.001		
4	0.560	0.648	0.136	<0.001		
5	0.554	0.678	0.182	<0.001		
6	0.553	0.664	0.167	<0.001		
7	0.577	0.678	0.149	<0.001		
8	0.569	0.667	0.147	<0.001		
9	0.591	0.685	0.137	<0.001		
10	0.605	0.722	0.161	<0.001		
11	0.554	0.662	0.163	<0.001		
12	0.571	0.694	0.178	<0.001		
13	0.586	0.675	0.132	<0.001		
14	0.558	0.660	0.155	<0.001		
15	0.529	0.654	0.192	<0.001		
16	0.590	0.685	0.138	<0.001		
17	0.518	0.654	0.208	<0.001		
18	0.593	0.693	0.144	<0.001		
19	0.593	0.648	0.085	0.076		
20	0.548	0.679	0.193	<0.001		
Median	0.571	0.676	0.148	–		
Minimum	0.518	0.648	0.085	–		
Maximum	0.605	0.722	0.208	–		

## Appendix C. Missing-Data Handling and Completed Datasets

This appendix provides additional details on the identification and treatment of missing values in the seven matching covariates defined in Section 3.3. Missingness was distinguished between questionnaire-form-related planned missingness and residual ordinary missingness. Only cells classified as planned missingness were designated for imputation; ordinary missing values were not imputed.

The questionnaire-form audit used the 29,350 students in the four national source samples with valid ESCS and PV1MATH values, before restriction to TICKET participants. Questionnaire forms 1, 2, and 3 contained 9846, 9773, and 9731 students, respectively. For forms with at least 10 observations, a variable–form combination was classified as planned missingness when its form-specific missing rate was at least 80% and exceeded the mean missing rate in the other eligible forms by at least 60 percentage points. This procedure identified TEACHSUP, STUDREL, and MTSUP as planned missing in form 1 and MMINS as planned missing in form 2 (Table A7). Missing values in these variable–form combinations were designated as planned missingness. Smaller missing percentages in administered forms were classified as ordinary missingness.

**Table A7:** Questionnaire-form missingness and planned-missingness classification.

Variable	Form 1 Missing (%)	Form 2 Missing (%)	Form 3 Missing (%)	Rotated	Planned Variable–Form Combination
TEACHSUP	100.000	0.522	1.079	Yes	TEACHSUP–form 1
STUDREL	100.000	0.460	1.459	Yes	STUDREL–form 1
MTSUP	100.000	0.696	1.583	Yes	MTSUP–form 1
MMINS	11.284	100.000	9.865	Yes	MMINS–form 2

Table A8 applies this classification to the 929 disadvantaged students linked to valid TICKET records. Planned cells were filled in each completed dataset, whereas ordinary missing values were not imputed. Students with at least one ordinary missing value among the seven matching covariates were excluded after the planned cells had been generated. Variable-specific ordinary-missing counts overlap and therefore cannot be summed to obtain the number of excluded students. After deduplication, the same 221 students were excluded, leaving 708 students, including 274 resilient and 434 non-resilient students, in every completed dataset.

**Table A8:** Missing-data counts and sample retention.

Variable	Total Missing	Planned Cells	Ordinary Missing	Cells Imputed per Dataset
ST04Q01	0	0	0	0
IMMIG	44	0	44	0
FAMSTRUC	97	0	97	0
TEACHSUP	323	317	6	317
STUDREL	323	317	6	317
MTSUP	328	317	11	317
MMINS	424	323	101	323

Twenty completed datasets were generated using a Bayesian-ridge-assisted donor imputation procedure within an iterative chained-equation structure (van Buuren & Groothuis-Oudshoorn, 2011). For each target variable, the imputation model included the other six matching covariates together with country, ESCS, PV1MATH, questionnaire form, and resilience status. Bayesian ridge regression was fitted using observations with an observed value of the target variable. For each planned-missing cell, one value was sampled from five observed donors whose fitted values were closest to a perturbed predicted value. Only cells classified as planned missingness were imputed; ordinary missing values were left unchanged. After students with residual ordinary missingness were excluded, the same 708 students were retained in each of the 20 completed datasets. Propensity-score estimation, matching, and balance assessment were then conducted separately in each completed dataset.

## Appendix D. Behavioral-Profile and OT–CSI Sensitivity Analyses

This appendix reports sensitivity analyses for both the behavioral-profile solution and the supplementary OT–CSI analysis. First, the behavioral-profile analysis was repeated using alternative LCS similarity thresholds to examine whether the profile–resilience association depended on the reference choice of κ=0.50. Second, the OT–CSI analysis was examined across alternative values of α and ϵ and against the pair-preserving permutation reference distribution.

Across the 15 combinations of α∈{0.3,0.4,0.5,0.6,0.7} and ε∈{0.02,0.03,0.04}, CSI ranged from 0.144 to 0.150 (Figure A2). The variation was small across the examined parameter settings, and CSI did not increase monotonically with α.

**Table A9:** Sensitivity of behavioral profile solutions across LCS similarity thresholds.

κ	No. of Profiles	ARI vs. 0.50	Largest Profile (%)	Profile–Resilience Statistic	Paired Permutation *p*	Reference-Pattern Retention
0.40	8	0.471	50.2	$\chi^{2}=34.970;\;V=0.282$	<0.001	91.2%
0.45	10	0.815	37.0	$\chi^{2}=44.012;\;V=0.316$	<0.001	98.6%
0.50	14	1.000	33.6	$\chi^{2}=43.151;\;V=0.313$	<0.001	Reference
0.55	20	0.748	29.1	$\chi^{2}=55.919;\;V=0.356$	<0.001	85.8%
0.60	27	0.563	22.0	$\chi^{2}=55.652;\;V=0.356$	<0.0001	83.1% across two split profiles

Across the five threshold specifications, the number of identified profiles ranged from 8 to 27. Agreement with the reference solution at κ=0.50 was highest at the adjacent thresholds of 0.45 and 0.55, with adjusted Rand indices of 0.815 and 0.748, respectively. The profile–resilience association remained statistically detectable under the pair-preserving permutation procedure at all five thresholds, with Cramér’s *V* ranging from 0.282 to 0.356. The principal resilience-enriched behavioral pattern was also retained across the examined thresholds, although the exact profile resolution changed as κ increased.
